# Spectroscopic, Thermal, Microbiological, and Antioxidant Study of Alkali Metal 2-Hydroxyphenylacetates

**DOI:** 10.3390/ma14247824

**Published:** 2021-12-17

**Authors:** Mariola Samsonowicz, Ewelina Gołębiewska, Elżbieta Wołejko, Urszula Wydro, Grzegorz Świderski, Joanna Zwolińska, Monika Kalinowska, Włodzimierz Lewandowski

**Affiliations:** 1 Department of Chemistry, Biology and Biotechnology, Institute of Civil Engineering and Energetics, Faculty of Civil Engineering and Environmental Science, Bialystok University of Technology, Wiejska 45E Street, 15-351 Bialystok, Poland; e.golebiewska@pb.edu.pl (E.G.); e.wolejko@pb.edu.pl (E.W.); u.wydro@pb.edu.pl (U.W.); g.swiderski@pb.edu.pl (G.Ś.); m.kalinowska@pb.edu.pl (M.K.); w-lewando@wp.pl (W.L.); 2 Centre for Advanced Technologies, Adam Mickiewicz University, Uniwersytetu Poznańskiego 10 Street, 61-614 Poznan, Poland; joakol1@amu.edu.pl

**Keywords:** 2-hydroxyphenylacetic acid, alkali metal salts, spectroscopy, antioxidant, antimicrobial

## Abstract

The structural, spectral, thermal, and biological properties of hydroxyphenylacetic acid and lithium, sodium, potassium, rubidium, and cesium 2-hydroxyphenylacetates were analyzed by means of infrared spectroscopy FT-IR, electronic absorption spectroscopy UV-VIS, nuclear magnetic resonance ^1^H and ^13^C NMR, thermogravimetric analysis (TG/DSC), and quantum-chemical calculations at B3LYP/6-311++G** level. Moreover, the antioxidant (ABTS, FRAP, and CUPRAC assays), antibacterial (against *E. coli*, *K. aerogenes*, *P. fluorescens*, and *B. subtilis*) and antifungal (against *C. albicans)* properties of studied compounds were measured. The effect of alkali metal ions on the structure, thermal, and biological properties of 2-hydroxyphenylacetates was discussed.

## 1. Introduction

**Figure 1:**
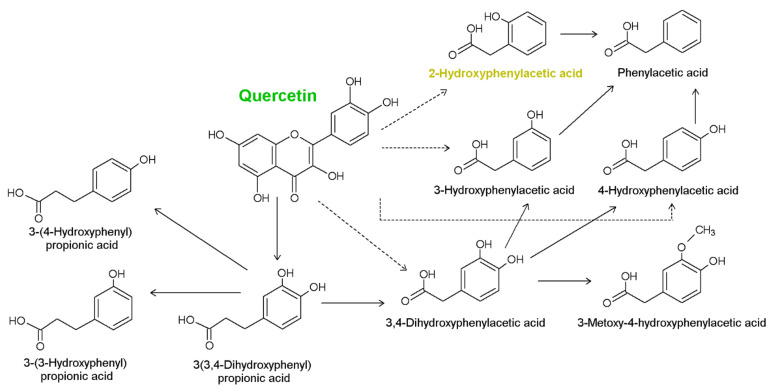
Possible pathways of quercetin degradation of by intestinal bacteria (on the basis of data from [6]).

Polyphenols are a wide group of natural bioactive substances found in many food sources, such as fruits, vegetables, cereals, legumes, spices, cocoa, and plant-derived beverages [1,2]. They are secondary metabolites of plants, involved in plant protection system against biotic (pathogens) and abiotic (e.g., exposure to cold, high temperatures, UV lights, drought, excessive water, salinity, or heavy metals) stress factors [3,4]. These compounds undergo biotransformation with the participation of bacterial enzymes. The phenolic acid metabolites, e.g., hydroxyphenylacetic or hydroxybenzoic acids, are formed in the colon, where they induce the physiologic effects [5,6]. Colon microflora degrades dietary phenolic compounds into small molecules, highly bioavailable metabolites such as hydroxyphenolic acids. Their biological activity (including antioxidant and microbiological) is often higher than the parent molecules. Figure 1 shows the possible pathways for the biodegradation of quercetin to hydroxyphenylacetic acids. 

Numerous studies have revealed that phenolic compounds found in the human diet play a key role in preventing various diseases related to oxidative stress, such as cancers, diabetes, and cardiovascular and neurodegenerative diseases [7,8,9,10,11]. These health benefits of polyphenols are strictly connected to their structure, namely the presence of phenol ring and the number and position of hydroxyl groups associated with the aromatic ring [12]. The antioxidant effect of phenolic compounds may consist in: donating of an electron or a hydrogen atom to neutralize the free radicals (reducing properties), quenching singlet oxygen, or binding metal ions such as Cu(II) and Fe(III) [13]. 

**Figure 2:**
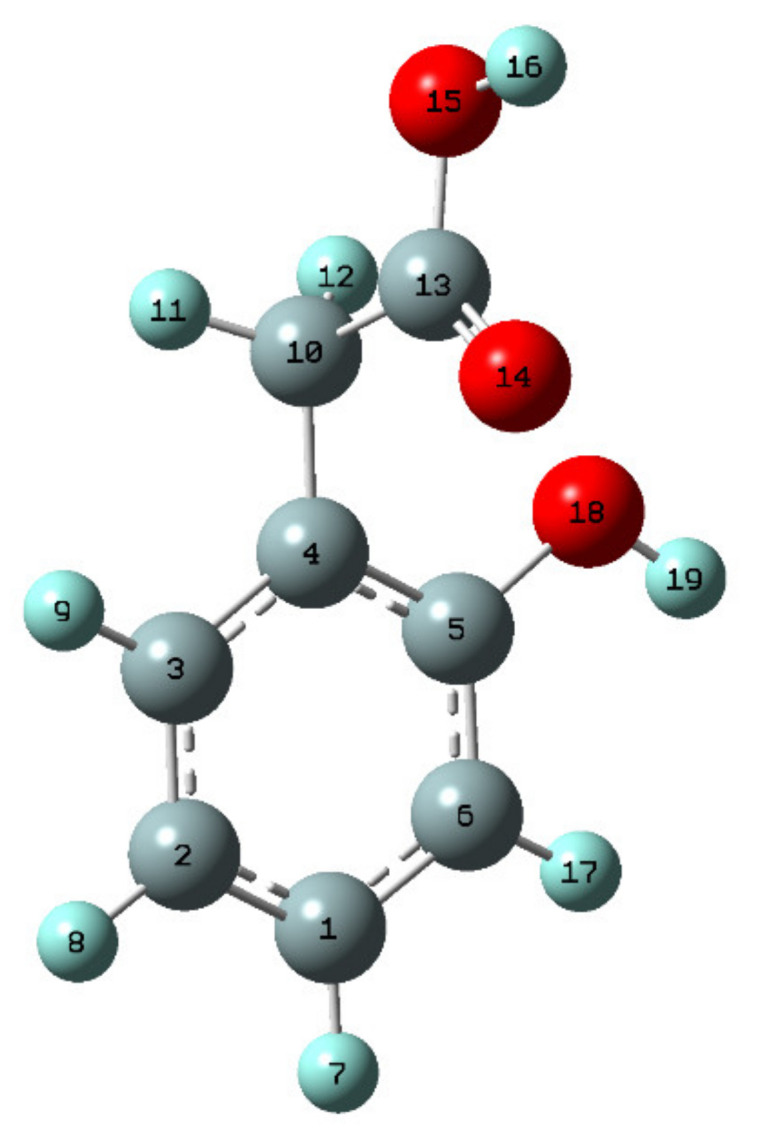
Chemical structure of 2-hydroxyphenylacetic acid (2-HPAA).

2-hydroxyphenylacetic acid (2-HPAA) (Figure 2) is a natural phenolic compound found in the genus *Astilbe* [14]. It is found in many foods in small amounts, including traditional medicinal food from the Tibetan plateau, for example: *Nitraria tangutorum Bobrov*, *Hippophae rhamnoides *L., *Lycium ruthenicum Murray*, *Lycium barbarum *L., and *Rubus corchorifolius *L.f. [15], fruits of *Vitex trifolia *L. *var. simplicifolia Cham.*, or herbs: *Vitex trifolia *L. (*Lamiaceae*) [16], oregano, burbot, wild leek, rice, orange mint, chanterelles, cascade huckleberries, garden tomato (var.), and grapes. [17,18]. This can make 2-hydroxyphenylacetic acid a potential biomarker for the consumption of these foods. It has been found that the amount of this acid depends on the part of the plant and its age [19]. Moreover, 2-HPAA is often used as an intermediate for the synthesis of bioactive products in pharmacy, e.g., antihypertensive agent [20]. 

The interest in naturally occurring antioxidants, which can be used as a substrate in dietary supplements, food, drugs, and cosmetics, has grown significantly over the past decade. In the literature, there are many reports on the increased or decreased antioxidant properties of the molecules after metal chelation [21,22,23]. Our previous studies indicated that there is a significant relationship between the molecular structure of the salts or metal complexes of phenolic compounds and their biological activity [21,22,23,24,25,26,27]. Metal ions can affect the biological properties of phenols, such as antioxidant and antimicrobial, by changing the electronic charge density in the molecules [26,27,28,29]. The results of our research showed that metal chelates can be characterized by higher antioxidant activity than their parent ligands [26,30,31,32], but not in every case [21,33]. In the study performed by Samsonowicz et al. [32], ursolic acid showed lower DPPH^•^ radicals scavenging activity than its complex with Cu(II) ions [32]. In other studies [27,30], the salts of chlorogenic and rosmarinic acid with lithium, sodium, and potassium showed better antioxidant and antimicrobial activity than the ligands alone. Moreover, an increase in the activity of alkali metal salts was observed in the systematic series: Li → Na → K (with increasing ionic radius) [27,30]. 

This paper is a part of a wide research topic the aimed at study of the influence of metals ions on the molecular structure, electronic system and bioactivity of the selected derivatives of benzoic and phenylacetic acids. We are interested in: what parameters of metal can influence the increase or decrease in the biological activity of ligands? In this work, the Li, Na, K, Rb, and Cs salts of 2-HPAA were synthesized and studied using experimental: FT-IR, UV-VIS, ^1^H, and ^13^C NMR spectroscopic, thermogravimetric (TG/DTG), and elemental analysis, as well as theoretical calculations at B3LYP/6-311++G(d,p) level using Gaussian program. The antioxidant activity of the synthesized salts was also studied and compared with the properties of ligand in the ABTS (2,2’-azino-bis(3-ethylbenzothiazoline-6-sulfonic acid), FRAP (ferric reducing antioxidant power), and CUPRAC (cupric ion reducing antioxidant capacity) assays. The microbiological activity of 2-HPAA and selected salts was tested against selected bacteria: *E. coli*, *K. aerogenes*, *P. fluorescens*, *B. subtilis*, and yeats *C. albicans*.

## 2. Materials and Methods

### 2.1. Materials

All reagents were of analytical purity and were used without further purification. 2-hydroxyphenylacetic acid, LiOH, NaOH, KOH, RbOH, CsOH, potassium persulfate (K_2_S_2_O_8_), copper(II) chloride (CuCl_2_), iron(III) chloride hexahydrate (FeCl_3_·6H_2_O), (2,2-azino-bis(3-ethylbenzothiazoline-6-sulfonic acid) diammonium salt (ABTS), ammonium acetate (CH_3_COONH_4_), 2,9-dimethyl-1,10-phenanthroline (neocuproine), sodium acetate trihydrate (C_2_H_3_NaO_2_∙3H_2_O), 2,4,6-Tris(2-pyridyl)-s-triazine (TPTZ), were purchased from Sigma-Aldrich Co. (St. Louis, MO, USA). Methanol was purchased from Merck (Darmstadt, Germany). 

### 2.2. Synthesis

All alkali metal salts of 2-hydroxyphenylacetic acid were obtained by mixing the weighed mass of 2-HPAA with an aqueous solution of appropriate metal hydroxides in a stoichiometric molar ratio 1:1. The resulting solutions were mixed and left to dissolve for 24 h at room temperature. Then, the remaining solvent was evaporated with a water bath and the obtained salts were dried at 105 °C for 24 h in a dryer. 

### 2.3. Spectroscopic Study

The FT-IR spectra of the solid samples in KBr pellets were recorded within 400–4000 cm^−1^ region on an Alfa spectrometer (Bruker, Billerica, MA, USA). The ultraviolet spectra were recorded in the range of 200–400 nm using the UV/VIS/NIR Agilent Carry 5000 spectrophotometer (Santa Clara, CA, USA). The ^1^H and ^13^C NMR spectra of 2-hydroxyphenylacetic acid and its salts were recorded with a Bruker Avance II 400 MHz. unit. All experiments were carried out in DMSO at room temperature using TMS (tetra methyl silane) as an internal reference.

### 2.4. Quantum-Chemical Calculations

To calculate optimized geometrical structures of the studied molecules the DFT (Density functional theory) hybrid method B3LYP/6-311++G(d,p) was used. Gaussian09 package of program was used to all calculations [34]. GaussView program was applied to visualize the results of theoretical calculations.

### 2.5. Thermogravimetric and Elemental Study 

The thermal stability and decomposition of 2-HPAA and its alkali metal salts were examined using a STA 600 Simultaneous Thermal Analyzer/FTIR Frontier (PerkinElmer, Waltham, MA, USA). The samples (5–6 mg) were heated in a ceramic crucible in the range 30–995 °C in flowing air atmosphere with a heating rate of 10 °C/min. The products of dehydration and decomposition processes were established on the basis of the TG, DSC, and DTG curves. The elemental analysis was conducted with the Perkin–Elmer 2400 Series II CHNS Analyzer. 

### 2.6. Antioxidant Properties 

The antioxidant activity of the 2-HPAA and its alkali metal salts was determined by the use of CUPRAC, ABTS^•+^, as well as FRAP assays. 

The ABTS radical scavenging method was performed according to Re et al. [35]. To obtain the ABTS^•+^ cation radical solution, the aqueous solution of ABTS (7 mM) and K_2_S_2_O_8_ (2.45 mM) were mixed in a 1:1 volume ratio and left for 16 h at room temperature (23 °C). An appropriate volume of methanol was added to the mixture (in volumetric ratio of 1:60) so that the obtained solution had an absorbance of about 0.7–1.0 at 734 nm. Furthermore, 1.5 mL of a methanolic solution of the tested compounds was added to 1 mL of the solution prepared in this way and, after 7 min of incubation, the absorbance was measured at a wavelength of 734 nm (in relation to methanol). Control samples contains methanol instead of tested compounds solutions. The antiradical activity against ABTS^•+^ cation radicals was presented as the percentage of their activity inhibition (%I):

(1) $$\%I=(\frac{A_{control}-A_{sample}}{A_{control}})\times 100\%$$

where: %*I*—Percent of inhibition of ABTS^•+^ cation radicals; *A_control_*—Absorbance of the control sample; and *A_sample_*—Absorbance of the tested sample. 

The FRAP assay was conducted following the method described by [36]. Briefly, the FRAP reagent was prepared by mixing acetate buffer (0.3 mM, pH 3.6) with TPTZ (10 mM, in 40 mM HCl) and FeCl_3_·6H_2_O (20 mM, in water), in a volumetric ratio 10:1:1. Then, 0.4 mL of methanolic solution of the tested substance (2.5 and 10 mM) was added to 3 mL of FRAP mixture, vortexed, and incubated for 8 min at room temperature. The absorbance was measured at 595 nm, against blank (i.e., 0.4 mL of methanol and 3 mL of FRAP reagent). The results were calculated and expressed as Fe^2+^ equivalents (µM) using the calibration curve prepared for FeSO_4_ (y = 3.4303x − 0.1058; R^2^ = 0.9984). 

The CUPRAC assay was performer according to [37]. To obtain the CUPRAC reagent, the aqueous solutions of CuCl_2_ (10 mM) and ammonium acetate (pH 7) were mixed with methanolic solution of neocuproine (75 mM), in a volumetric ratio 1:1:1. Then, 3 mL of this mixture was added to 0.5 mL of methanolic solution of the tested substance (100 and 250 mM) and 0.6 mL of distilled water. After 1 h of incubation at room temperature, absorbance was recorded at 450 nm against blank (methanol was used against sample). The results were expressed as Trolox equivalents (µM) using the calibration curve prepared for Trolox (y = 4.5758x + 0.0271; R^2^ = 0.9919). 

### 2.7. Cytotoxic Properties

*Escherichia coli* (ATCC 25922), *Klebsiella aerogenes* (ATCC 13048), *Pseudomonas fluorescens* (ATCC 13525), *Bacillus subtilis* (ATCC 11774), and *Candida albicans* (ATCC 10231) were obtained from the American Type Culture Collection (Manassas, VA, USA). Studied strains of bacteria were grown overnight in Mueller Hinton II Broth at 37 °C (E. coli, *B. subtilis*, and *K. aerogenes*) and at 26 °C (*P. fluorescens* and *C. albicans*). Then, the overnight cultures were diluted in fresh MH II Broth to obtain 10^8^ CFU/mL (CFU—colony forming units). For the antimicrobial activity was used the inoculum where the suspension of *E. coli*, *K. aerogenes*, *P. fluorescens*, and *B. subtilis* cells was 10^6^ CFU/mL and *C. albicans* cells was 104 CFU/mL. 

The preparation of two-fold dilution of tested substances has been performed according to Jabłońska-Trypuć et al. [38]. The final concentrations of analyzed compounds in each well were: 10 mM, 5 mM, 2.5 mM, 1.25 mM, 0.63 mM, 0.31 mM, and 0.16 mM. 2-HPAA and its alkali metal salts were dissolved in Tris-HCl and made up with double-distilled water. Antibacterial and antifungal activity of 2-HPAA and selected alkali metal salts, against *K. aerogenes*, *E. coli*, *B. subtilis*, *P. fluorescens*, and *C. albicans*, was estimated using MTT assay. The details were described previously [39]. The microbiological activity was presented as a relative cell viability of *E. coli*, *K. aerogenes*, *B. subtilis*, *P. fluorescens*, and *C. albicans* as compared to control and expressed in percentage. The determinations of antibacterial and antifungal activity in all samples were performed in triplicate. Ampicillin and kanamycin were used as a reference antibiotics.

## 3. Results

### 3.1. Spectroscopic Study 

The FT-IR spectra of 2-HPAA and its Li, Na, K, Rb, and Cs salts were presented in Figure S1. The wavenumbers, intensities, and assignment of the selected bands from the spectra of these compounds were gathered in Table 1. In the spectra of 2-HPAA, characteristic band assigned to the stretching vibrations of the carbonyl group ν(C=O) was found at the wavenumber: 1694 cm^−1^. The disappearance of this band in the IR spectra of 2-HPAA salts demonstrates that the alkali metal is bond to the 2-hydroxyphenylacetic acid through the carboxylate group. In this case, the ν(C=O) mode in the IR spectra of the 2-HPAA were replaced by the asymmetric and symmetric vibrations of the carboxylate anion ν_as_(COO^−^), ν_s_(COO^−^), as well as β_as_(COO^−^), and β_s_(COO^−^) in the IR spectra of metal salts. The intense bands of ν_as_(COO^−^) were in the range of 1564–1575 cm^−1^, ν_s_(COO^−^) in the region of 1381–1407 cm^−1^, β_as_(COO^−^), and β_s_(COO^−^) were located in the range of 633–693 and 865–882 cm^−1^, respectively. The bands assigned to bending asymmetric in-plane vibrations β_as_(COO^−^) moved towards lower wavenumbers in the series Cs > Rb = K > Li > Na 2-hydroxyphenylacetates (Table 2). The differences in the wavenumber of bands derived from the carboxylate group ν_as_(COO^−^), ν_s_(COO^−^), β_as_(COO^−^), and β_s_(COO^−^) did not change regularly with the increase in the atomic mass of the alkali metal ions. More regular changes were observed for previously reported phenylacetates and 3-hydroxyphenylacetates [24,40]. For example, the differences between wavenumbers assigned to the bands associated with the symmetric and asymmetric stretching vibrations of COO^-^ group (∆ν = ν_as_−ν_s_) changed in the series as follows: Li < Na < K > Rb < Cs 3-hydroxyphenylacetates and in the case of phenylacetic acid: Li < Na < K = Rb < Cs phenylacetates.

The bonding of metal ion to the carboxylic group in the place of hydrogen atom resulted in the disappearance of bands associated with the: stretching vibrations ν(OH) found at 3372 cm^−1^, in plane bending deformation β(OH) at 1385 cm^−1^, out of plane γ(OH) bending deformations of the hydroxyl group at 935 cm^−1^, in plane bending deformations β(C=O) at 783 cm^−1^, and out of plane γ(C=O) bending deformations of the carbonyl group at 622 cm^−1^ in the spectra of 2-HPAA salts. 

**Figure 3:**
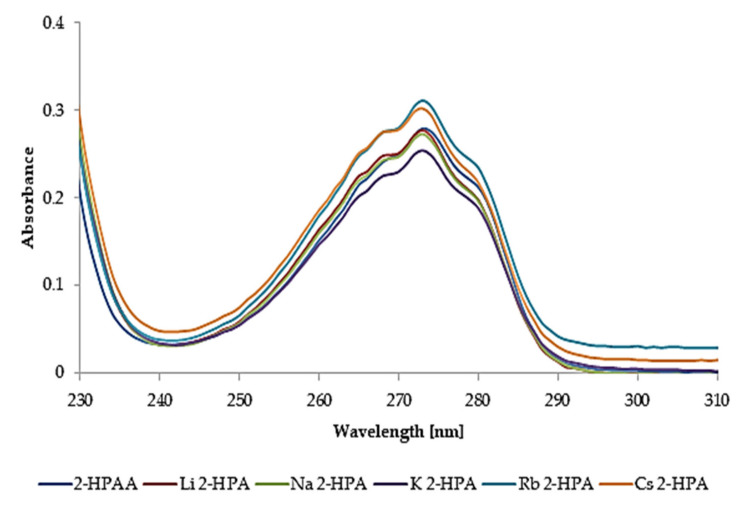
The UV spectra of the 2-HPAA and its alkali metal salts in methanol (0.1 mM).

Figure 3 presents the UV spectra of methanolic solutions of 2-HPAA and its alkali metal salts. No shifts or changes in the absorption maximum were found in the obtained spectra (λ_max_ = 273 nm). 

The values of the proton ^1^H NMR and carbon ^13^C NMR chemical shifts were summarized in Table S1. The proton spectrum of 2-hydroxyphenylacetic acid (Figure S2) showed: four signals located in the range of 6.76–7.12 ppm were assigned to the aromatic protons, a single signal derived from the aliphatic protons (CH_2_ group) (located at the 3.53 ppm), and signals assigned to the protons of hydroxyl groups: signal located at 9.44 ppm assigned to the -OH group of the carboxylic group, and the signal located at 12.13 ppm assigned to the proton of the hydroxyl group attached to the aromatic ring. The signal from the proton of the hydroxyl group of the aromatic ring is shifted downfield (above 12 ppm) due to the presence of hydrogen interactions of the hydroxyl group with the carbonyl atom in the acid and salts. On the FTIR spectra, bands of the hydroxyl group (aromatic ring) were observed below 3500 cm^−1^, which may indicate that the proton of this group forms hydrogen bond. In the ^1^H NMR spectra of the alkali metal salts of 2-hydroxyphenylacetic acid no signals from the protons of the carboxylic group were observed. The lack of a signal from a proton from the -COOH group proves that the metal is bind to the analyzed acid through the carboxylic group. The changes in the chemical shifts of aromatic protons towards the higher field were observed due to the substitution of alkali metal ions in the carboxylic group of the acid. In the ^1^H NMR spectra of 2-hydroxyphenylacetates a decrease in the chemical shifts of protons H7, H8, H9, and H17 compared to acid was noted. A decrease in chemical shifts usually indicates an increase in the screening the aromatic ring protons as a result of a circular current weakening [42]. Moreover, this proves the destabilization of the aromatic system of the salt compared with ligand and, according to the literature data, it may suggest a decrease in their aromaticity [43]. The signal values from the aliphatic protons of the CH_2_ group were also shifted towards the higher field. In the ^13^C NMR spectra of alkali salts (Figure S3), the greatest changes in the chemical shifts of the carbon atoms were noticed for the carbon from the aliphatic CH_2_ group and carboxylate group compared with the spectrum of ligand. Slightly smaller changes were observed in the case of the carbons of the aromatic system (comparing the ^13^C NMR spectra of HPAA and its alkali metal salts). 

### 3.2. Quantum-Chemical Calculations

In order to determine the influence of metals ions (Li, K, and Na) on the electronic structure of the 2-HPAA, the geometry optimizations of ligand and selected salts were carried out by the DFT method using the B3LYP/6-311++ G(p,d) basis set. 

The selected bond lengths and angles between bonds in 2-HPAA and alkali metal 2-hydroxyphenylacetates were presented in Table S2 (those listed which changed their value by 0.005 Å. and 1° comparing 2-HPAA and its alkali metal salt molecules). The atom numbering scheme adopted in this study was given in Figure 2. Substitution of the alkali metal ions to the carboxylic group of 2-HPAA significantly changed the lengths of some bonds in the acid molecules. It was shown from the comparison between the optimized geometry structures of 2-HPAA and its metal salts that the lengths of the C10-C13 and C13-O14 bonds lengthened by 0.010–0.024 Å and 0.058–0.065 Å, respectively, upon salts formation. Moreover, a significant extension in the bond length between the O15-H16/M atoms was observed (in the range of 0.884 to 1.545 Å compared to the 2-HPAA molecule). In the case of C13-O15 bond, the shortening of the lengths by 0.086–0.092 Å, in comparison to the acid molecule, was noticed. The lengths of the C10-C13 and O15-H16/M bonds increased with increasing atomic weight of the alkali metal (in following order: 2-HPAA < Li 2-HPA < Na 2-HPA < K 2-HPA), while the lengths of the C13-O15 bond decreased, also in the same series. 

Taking into account the values of angles, the increase of 0.4–1.01°, 4.48–4.71°, and 6.04–7.57° for C3-C4-C5, C4-C10-C13, and C10-C13-O14 atoms, respectively, and the decrease of 27.37–35.97° for C13-O14-H16/M was observed for salts compared with acid molecule. The values of angles between these atoms changed regularly in following order: 2-HPAA < Li·2-HPA < Na·2-HPA < K·2-HPA. This showed that the structure of carboxylic group was mostly during the salt formation process.

**Figure 4:**
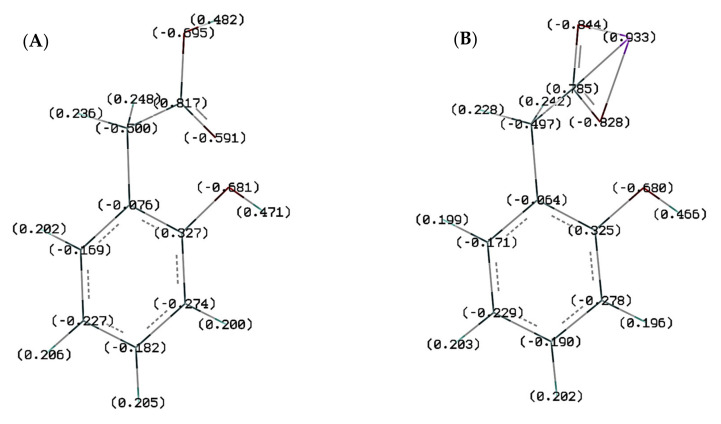

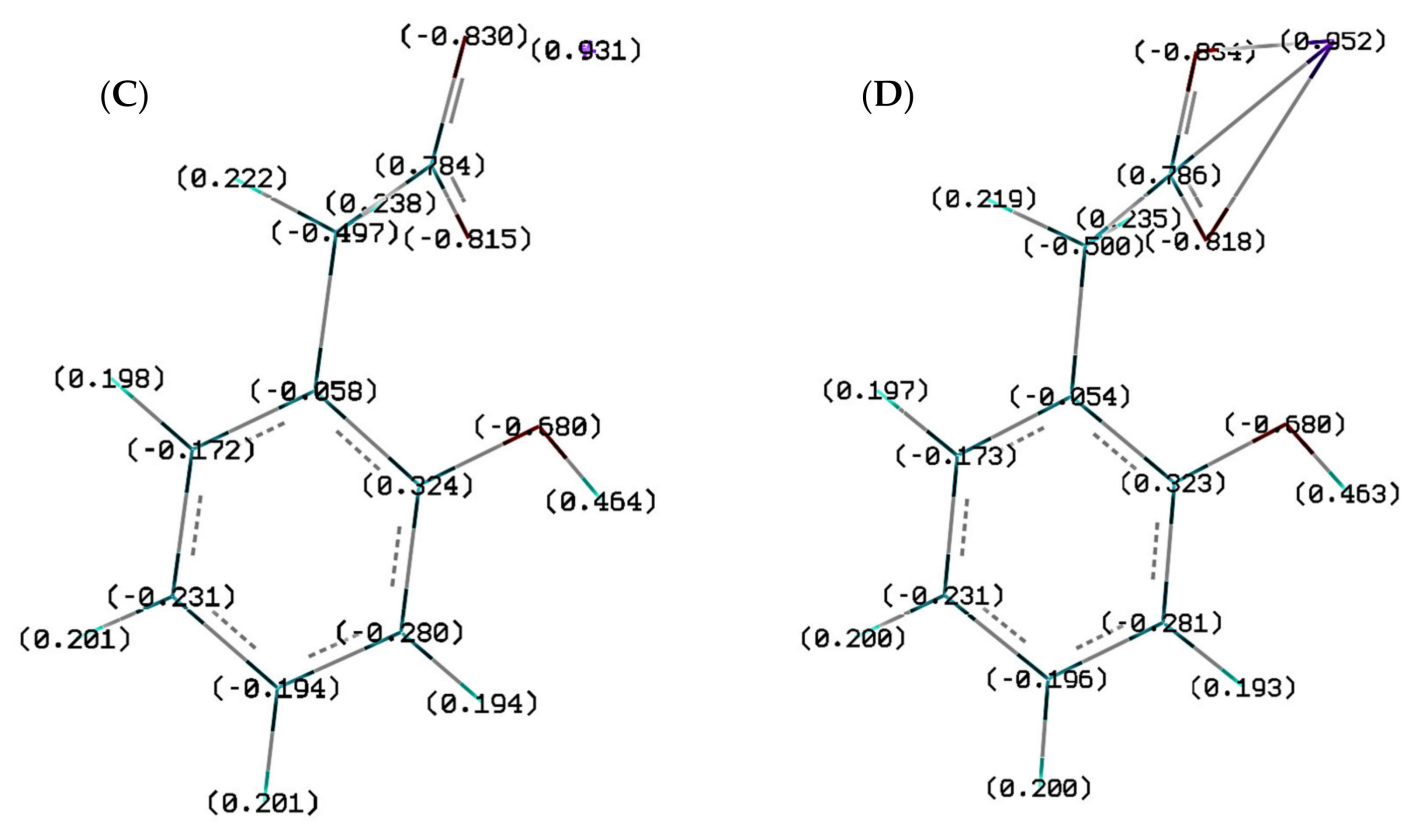
Electronic charge distribution in optimized molecules of (**A**) 2-HPAA, (**B**) Li 2-HPA, (**C**) Na 2-HPA, and (**D**) K 2-HPA calculated by NBO method.

The NBO (Natural Bond Orbital) and Mulliken atomic charges calculated by methods B3LYP/6-311++G** were collected in Table S3. After replacing the hydrogen in the carboxylic group with a metal ion (Li, Na, and K), there was a change in the distribution of electronic charge around some atoms. In the aromatic ring around the C1, C2, and C4 carbon atom there was a decrease in the negative charge, and around the C3 carbon atom this charge increased. In the –CH_2_ group around the hydrogen atoms, the electron charge decreased in following order: 2-HPAA < Li 2-HPA < Na 2-HPA < K 2-HPA. The electron charge distributions calculated by the NBO method were shown in Figure 4. 

The total electronic charge density in the carboxylate anion -COO^−^ was also analyzed and the results were presented in Table S4. Both the NBO and Mulliken methods confirmed that the substitution of the metal in the carboxylic group increased the total value of the negative charge on the -COO^−^ anion. In the case of Milliken’s method, a regular increase in this charge can be observed in the following sequence: 2-HPAA < Li 2-HPA < Na 2-HPA < K 2-HPA. The values of the electronic charge calculated in the NBO method did not meet this regularity, however, it can be noticed that the values obtained for 2-HPAA alkali metal salts were higher than for the acid itself. 

The calculated values of the dipole moments (Table 3) of 2-HPAA and its chosen alkali metal salts showed that their polarity increased with the increase in the atomic mass of the metal (2-HPAA → Li 2-HPA → Na 2-HPA → K 2-HPA). The values of absolute energy of tested molecules increased in reverse order (K 2-HPA → Na 2-HPA → Li 2-HPA → 2-HPAA). 

**Figure 5:**
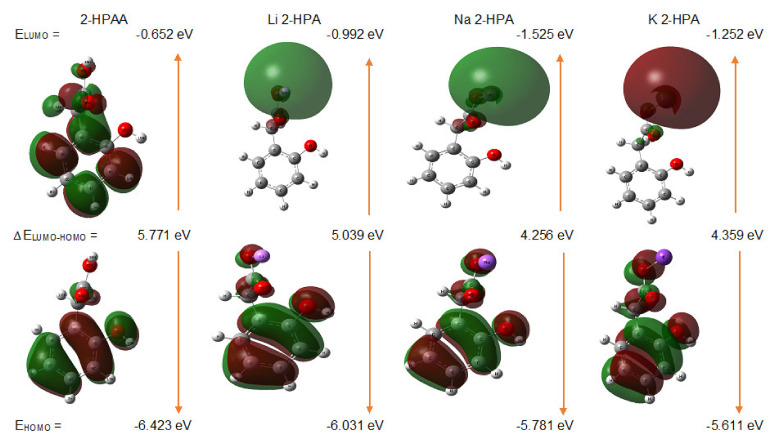
The atomic orbital components of the frontier molecular orbital of 2-hydroxyphenylacetic acid (2-HPAA) and their lithium (Li 2-HPA), sodium (Na 2-HPA), and potassium (K 2-HPA) salt.

The energy values of the highest (HOMO) and lowest (LUMO) unoccupied molecular orbitals can be used to assess the chemical and biological activity of molecules [25,27]. Figure 5 presented the energy and distribution of the HOMO and LUMO orbitals obtained for the optimized structures of the 2-HPAA and its chosen alkali metal salts calculated at DFT/B3LYP/6-311++G(d,p) level. The energy of the HOMO orbital characterized the electron-donating character of a compound, while the energy of the LUMO orbital was related to the ability to accept electrons [44]. The HOMO orbital for 2-HPAA molecule was largely distributed over the aromatic ring, -CH_2_ group, and -OH group (in the carboxyl group), while the LUMO orbital was distributed over the benzene ring, -CH_2_ and -COOH groups. In the case of 2-hydroxyphenylacetates, the HOMO orbitals were located almost on the entire structure, while the LUMO orbitals were mostly located on the alkali metal ion: Li, Na, and K. HOMO orbital energy for all analyzed 2-hydroxyphenylacetates increased compared to 2-HPAA molecule. The HOMO energies values increased as follows: 2-HPAA < Li 2-HPA < Na 2-HPA < K 2-HPA. The red and green color distributions represent positive and negative phase in molecular orbital wavefunction, respectively [45]. In the case of K 2-HPA molecule, a high electron density was observed within the potassium ion (red color) in LUMO orbital.

Based on the energies of the HOMO and LUMO orbitals, general reactivity descriptors such as energy gap (ΔE_(LUMO-HOMO)_), ionization potential (IP = −E_HOMO_), electronaffinity (A = −E_LUMO_), electronegativity (χ = (IP+A)/2), electronic chemical potential (μ = −(IP+A)/2), chemical hardness (η = (IP-A)/2), chemical softness (σ = 1/(2∙η)), and electrophilicity index (ω = μ^2^/2), were calculated and gathered in Table 3. The value of the ∆E characterizes the chemical reactivity, bioactivity, and kinetic stability of the compound [46]. In our study, the greatest difference between the energies of the HOMO and LUMO orbitals (ΔE) was shown for 2-HPAA (5.771 eV), while the smallest for Na 2-HPA (4.256 eV). This indicates that Na 2-HPA has the highest chemical reactivity and the lowest kinetic stability for all studied molecules. Moreover, alkali metal salts showed higher values of HOMO energy and lower values of energy gap than the ligand. It indicates that the chosen metal salts are more reactive antioxidants than 2-HPAA. 

The HOMO–LUMO energy gap for 2-HPAA (5.7710 eV) was lower than for phenylacetic acid (6.64 eV) [47] and higher than for 3-HPAA (4.243 eV) [24]. The obtained results indicate that the presence and position of the hydroxyl group in the structure of phenylacetic acids affect their biological activity. 2-HPAA shows much weaker antibacterial activity compared to 3-HPAA which possess the hydroxyl group from the ring in the meta position. 

Chemical hardness and softness determine the stability and chemical activity of the compound, respectively. Stable compounds are characterized by higher hardness, while highly reactive compounds have high softness value [48]. The chemical hardness of studied compounds increased in the series: Na 2-HPA → K 2-HPA → Li 2-HPA → 2-HPAA, while the softness increased in the reverse series. A similar trend was observed in the case of previously reported 3,4-dihydroxyphenylacetates [25] The higher the electrophilicity index, the higher the molecule’s ability to receive electrons. Among the screened compounds, the highest ω value showed Na 2-HPA (3.1354 eV), while the lowest was observed for 2-HPAA (2.1684 eV).

### 3.3. Thermogravimetric Study

**Figure 6:**
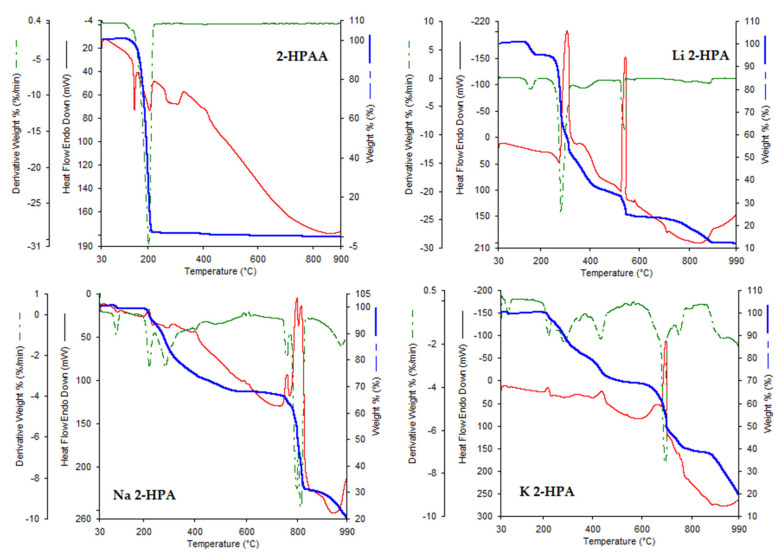

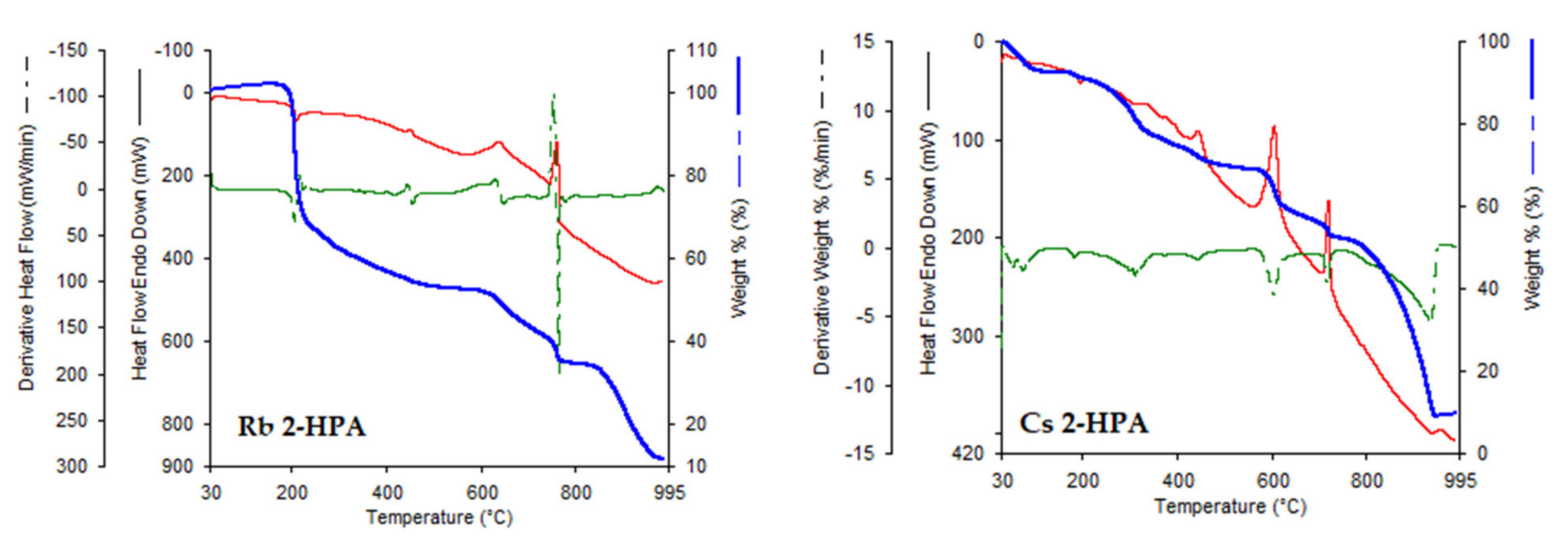
The DSC (red line) and TG (blue line)/TDG (green line) curves of 2-HPAA and its alkali metal salts obtained in air atmosphere.

The results of elemental and thermogravimetric analyses of 2-HPAA and its selected alkali metal salts were shown in Figure 6 and Table 4 and Table 5. The TG/DTG curves indicated that the completely thermal decomposition of 2-HPAA occurred in one step in the temperature range 130–217 °C, and with the total weight loss about 98.49%. The DSC curve for pure 2-HPAA showed a sharp endothermic transition at 150.99 °C which was related probably to decarboxylated of acid. Then, there was complete thermal decomposition of the acid without end products. 

Thermal decomposition of alkali metal salts with 2-hydroxyphenylacetic acid took place in several stages. The first step was thermal dehydration in the case of lithium and cesium salts. The remaining salts were anhydrous. In the next stage of thermal decomposition, thermal degradation of the phenolic ring occurred. In this stage, dehydroxylation of the aromatic ring took place, as evidenced by the endothermic transition peaks on the DSC curve. In the next stages, we observed the peaks of exothermic changes related to the decomposition of the aromatic ring. Thermal decomposition of salt led to alkali acetates and then to alkali carbonates. In the case of thermal decomposition of the lithium salt, the end product was lithium oxide Li_2_O, and in the case of rubidium salt, rubidium oxide Rb_2_O. In the remaining case, the final products of the thermal transformation were not determined. In the studied temperature range, the sodium, potassium and cesium salts decomposed into carbonates, and their further thermal decomposition occurred (the products was not specified). Among the tested alkali metal salts, the most thermally stable were the potassium and lithium salts, the decomposition of which starts at about 200 °C. The thermal decomposition of the remaining salts begun at about 116 °C (sodium salt), 127 °C (cesium), and 160 °C (rubidium). Compared to the acid, the tested salts showed higher thermal stability. Thermal decomposition of acid started from about 110°C. 

Table 5 presents the results of the elemental analysis of the tested salts. Analysis showed that the metal was combined with the ligand (2-hydroxyphenylacetic acid) in a molar ratio of 1:1. The lithium salt was hydrated and contained half the water molecule, which was confirmed by the results of the thermal analysis. 

### 3.4. Antioxidant Activity 

**Figure 7:**
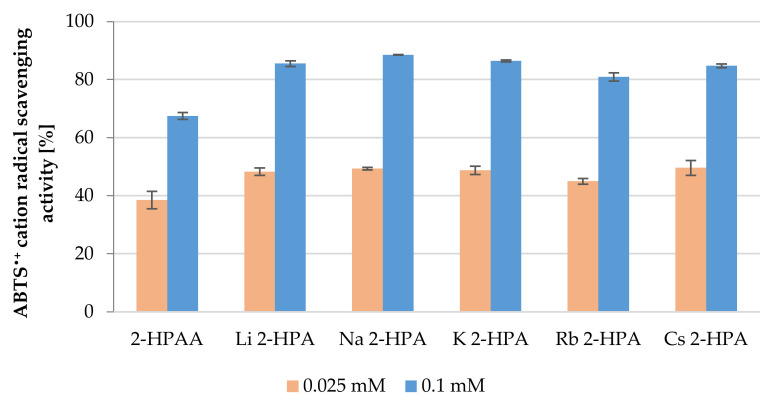
Antioxidant properties of the 2-HPAA and its alkali metal salts expressed as percentage of ABTS^•+^ cation radicals inhibition (concentrations of tested substances in the samples 0.025 and 0.01 mM).

The results of reactions of 2-HPAA and its alkali metal 2-hydroxyphenylacetates with ABTS^•+^ cation radicals were presented as the percent of inhibition of ABTS^•+^ radicals (Figure 7). Alkali metal salts were better scavengers of cation radicals than 2-HPAA. Moreover, the antiradical activity of 2-HPAA and its alkali metal 2-hydroxyphenylacetates measured in the ABTS assay increased with the increase in their concentration. At a concentration of 0.025 mM, 2-HPAA inhibited 38.55% of the initial concentration of ABTS^•+^ radicals, while its alkali metal salts inhibited from 44.98 to 49.62% of radicals. The antioxidant effect of the salts of 2-HPAA (0.1 mM) increased in the following series: Rb → Cs → Li → K → Na HPA. In the concentration of 0.1 mM, the highest value of ABTS^•+^ radicals inhibition was observed for Na 2-HPA (88.51 ± 0.10%), whereas the lowest inhibition value was recorded for 2-HPAA (67.49 ± 1.20%). 

**Figure 8:**
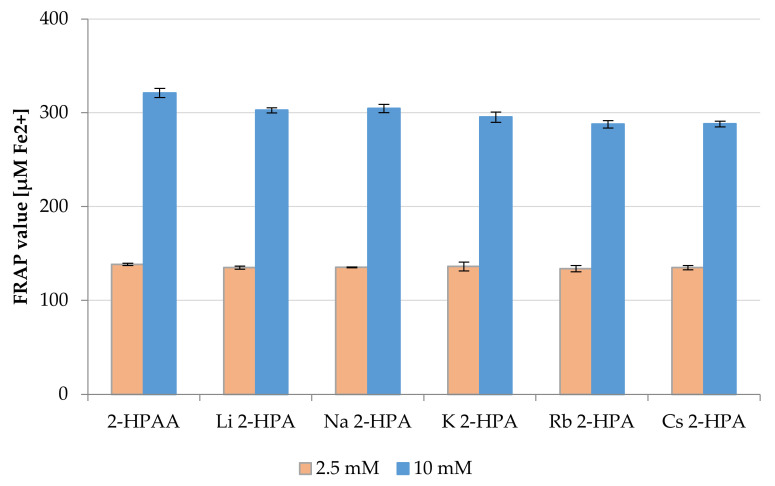
Antioxidant properties of the 2-HPAA and its alkali metal salts expressed as FRAP value (μM Fe^2+^) (concentrations of tested substances in the samples 2.5 and 10 mM).

The results of FRAP assay demonstrated that 2-HPAA and its alkali metal 2-hydroxyphenylacetates have ferric reducing antioxidant activity (Figure 8). The FRAP value of tested compounds ranged from 133.88 to 138.31 μM Fe^2+^ for the concentration of 2.5 mM, and from 287.87 to 321.13 μM Fe^2+^ for the concentration of 10 mM. In this assay, at a lower concentration of substance (2.5 mM), all studied compounds showed similar antioxidant activity. At the concentration of 10 mM, slowly higher antioxidant activity was obtained for 2-HPAA. The antioxidant activity of the tested salts (10 mM) can be arranged in the following series: Rb ≤ Cs < K < Li < Na. The FRAP values showed that all the tested compounds had a very small ferric reducing antioxidant power (FRAP) compared with natural antioxidant–L-ascorbic acid. The reducing power of L-ascorbic acid in the same conditions at the concentration of 50 μM was 155.43 μM Fe^2+^. While similar FRAP values for 2-HPAA and its salts were obtained for the concentration of these compounds of 2.5 mM. The results obtained in the CUPRAC assay indicated the same tendency

**Figure 9:**
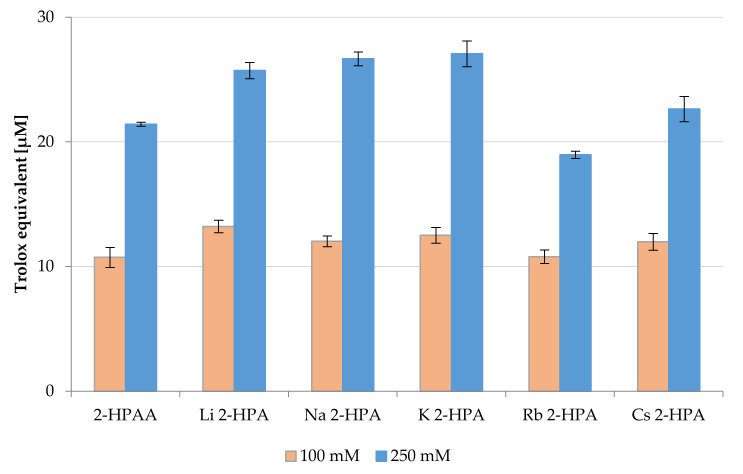
Antioxidant properties of the 2-HPAA and its alkali metal salts expressed as Trolox equivalents (μM) (concentrations of tested substances in the samples 100 and 250 mM).

The results of CUPRAC assay were presented in Figure 9. The obtained CUPRAC value for tested compounds ranged from 10.74 to 13.21 μM of Trolox for the concentration of samples 100 mM, and from 18.97 to 27.07 μM of Trolox for 250 mM samples. The highest ability to reduce copper(II) ions were observed for Li 2-HPA, Na 2-HPA, and K 2-HPA (at a concentration of 250 mM) 25.72, 26.66, and 27.07 μM of Trolox, respectively. The activity of the 2-HPAA salts (250 mM) increases in the series: Rb → Cs → Li → Na → K. In the case of 100 mM samples, no significant differences were found. 

Generally, the results of ABTS and CUPRAC assays showed that the tested alkali metal salts have better antioxidant properties than the acid alone, but no such dependency was estimated on the basis of FRAP assay. The differences in the above results may result from differences in the mechanisms of action of the conducted tests. ABTS assay is based on a combination of SET (single electron transfer) and HAT (hydrogen atom transfer) mechanisms, whereas FRAP and CUPRAC assays are based only on the SET mechanism [31]. In addition, differing times of reaction and kinetics between the tested compound and reagent may cause differences in the results of individual tests [49]. There are many reports in the literature about the influence of alkali metals on the biological activity of the parent phenolic acid. For example, study of Świsłocka et al. [27], demonstrated that alkali metal (Li, Na, K) salts of rosmarinic acid, showed higher antioxidant activity in DPPH^•^ and FRAP assays than acid alone [27]. Similar dependence was obtained in our study, lithium, sodium, and potassium salts of 2-HPAA showed the highest antioxidant activity in the ABTS, FRAP, and CUPRAC assays. The antioxidant and anti-inflammatory activities of plant extracts containing 2-HPAA were reported as well [50,51,52]. 

### 3.5. Antibacterial and Antifungal Activity 

**Figure 10:**
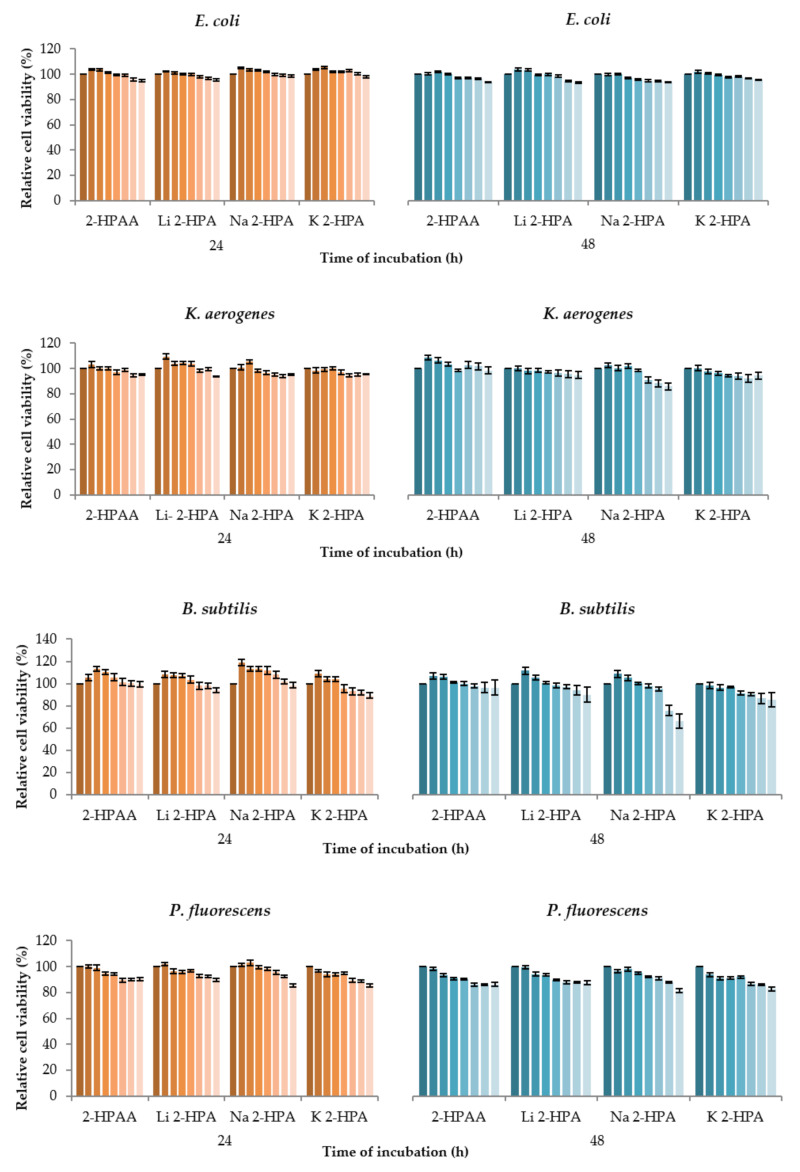

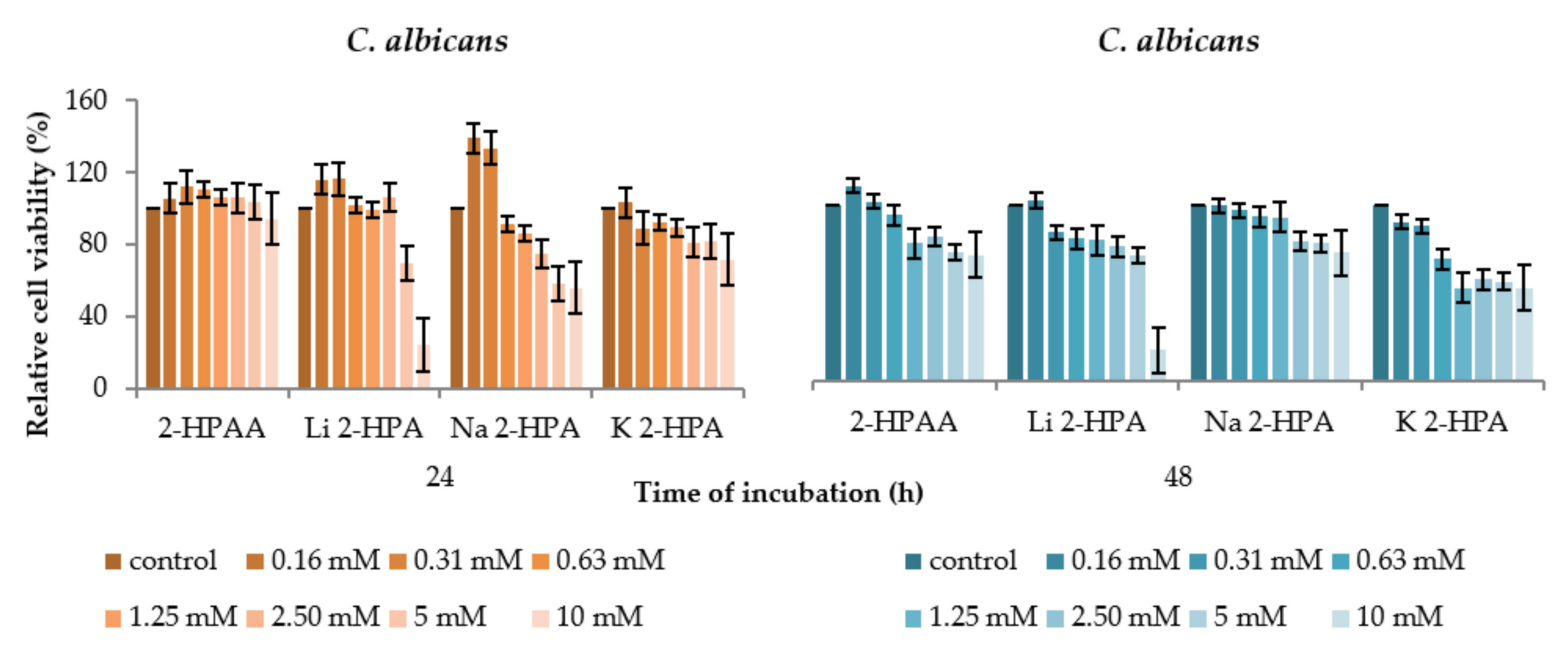
Cytotoxicity of 2-HPAA and its alkali metal salts with Li, Na, and K on bacteria (*E. coli*, *K. aerogenes*, *B. subtilis*, and *P. fluorescens*) and fungal (*C. albicans*) strains expressed as relative cell viability (%) compared to the control sample (untreated cells—100%).

**Figure 11:**
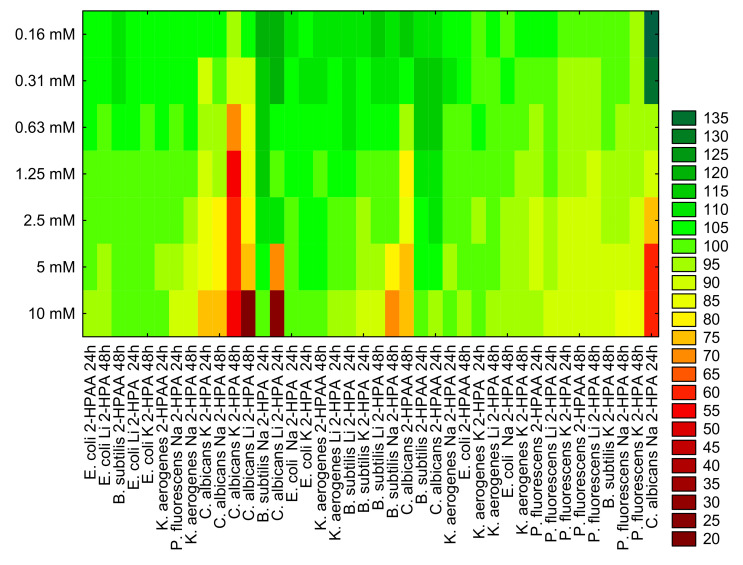
Cluster analysis grouping objects into similar groups based on relative bacteria (*E. coli*, *K. aerogenes*, *B. subtilis*, and *P. fluorescens*) and fungal (*C. albicans*) cells viability (%) after treatment of 2-HPAA and its alkali metal salts with Li (Li-2-HPA), Na (Na-2-HPA), and K (K-2-HPA) and their different concentration (0.16 mM, 0.31 mM, 0.63 mM, 1.25 mM, 2.5 mM, 5 mM, and 10 mM) after 24h and 48h of incubation.

Antibacterial and antifungal activity of 2-HPAA, Li 2-HPA, Na 2-HPA, and K 2-HPA were tested against microbial (*E. coli*, *K. aerogenes*, *B. subtilis*, and *P. fluorescens*) and fungal (*C. albicans*) strains. The results were compared with a control sample (i.e., untreated cells—100%) and expressed as % of live cells (Figure 10 and Figure 11). Generally, the cell viability decreased with increasing concentration of tested compounds. Incubation of *C. albicans* with Li 2-HPA led to the greatest decrease in cell viability. At the highest tested concentration (10 mM), Li 2-HPA caused a decrease in relative cell viability—about 76% and 82% after 24 and 48 h treatment, respectively. Other tested compounds at the same concentration reduced the relative cell viability in *C. albicans* from 44% to 6% after 24 h of incubation, and from 47% to 27% after 48 h. Interestingly, at a lower concentration of K 2-HPA, i.e., from 5 mM to 0.63 mM, a reduction in cell viability by ~47% to 30% (after 48 h of incubation) was observed compared to the control sample. 2-HPAA showed no significant effect on the viability of *C. albicans* fungal cells after 24 h, while after 48 h, a decrease in viability was observed by 27% at the concentration of 5 mM and by 21% at the concentration of 1.25 mM. The application of 2-HPAA and its tested alkali metal salts, in all analyzed concentrations, on *E. coli* and *K. aerogenes* did not cause any significant decrease in the relevant viability of bacterial cells, with one exception. In the case of *K. aerogenes*, incubated for 48 h with Na 2-HPA, the small decrease in relative cell viability was observed at 5 mM and 10 mM, by about 12% and 14%, respectively. Other tested compounds at the same concentrations reduced the viability of *K. aerogenes* by less than approximately 8%. In addition, Na 2-HPA also caused the greatest changes in cell viability of *B. subtilis* compared to other tested compounds. After 48 h, at the highest tested concentrations (10 mM and 5 mM), Na 2-HPA was found to reduce the cell viability of *B. subtilis* by about 34% and 24%, respectively. The analyzed compounds decreased the relative viability of *P. fluorescens* cells, and the degree of this reduction depended on the concentration of the compounds (although slight changes were observed). For example, after 48 h, 2-HPAA at 0.16 mM caused 2% decline in *P. fluorescens* cell viability, at 1.25 mM by 10% and at 2.5 mM by 14% compared to the control untreated cells. 

Summarizing, 2-HPAA and its alkali metal salts with Li, Na, and K in tested concentrations (0.16–10 mM) did not show significant antibacterial properties on the tested bacteria (*E. coli*, *K. aerogenes*, *B. subtilis*, and *P. fluorescens*). Only slight declines in bacterial survival were noted. While the reference antibiotics significantly inhibited microbial growth (about 100%) at much lower concentrations: (0.43–0.57 and 0.21 mM for ampicillin and kanamycin, respectively). However, in our study, tested salts were found to be moderate antifungal agents. These compounds, at the highest tested concentration (10 mM), inhibited *C. albicans* cell growth by about 76%, 44%, and 28% after 24 h and 82%, 27%, and 47%, respectively for Li 2-HPA, Na 2-HPA, and K 2-HPA. Unfortunately, antimicrobial effects of 2-HPAA and tested salts are very limited in the literature, e.g., in the work of Chapla et al. [51], 2-HPAA isolated from endophytic fungus *Colletotrichum gloeosporioides* exhibited high antifungal activities against *C. cladosporioides* and *C. sphaerospermum* fungal strains (using thin-layer chromatography (TLC) diffusion method) [52]. However, there are several reports about the influence of the other carboxylic acids and its salts on microorganisms. For example, Ozdemir and Soyer [53], reported that 3-hydroxyphenylacetic acid (3-HPAA) has a dose-dependent antibacterial effect on *P. aeruginosa*. The percentage of inhibition of bacterial growth increased with increasing concentration of 3-HPAA, e.g., 3-HPAA at concentration of 1.9 mg/mL showed a 58% inhibition, while at 2.1 mg/mL it showed 98%. Moreover, incubation of *P. aeruginosa* with 3-HPAA at a concentration of 2.3 mg/mL did not result in any persistent bacteria [53]. Yujia Liu et al. described the inhibitory effect of 4-phenylacetic (4-HPCA) acid on *L. monocytogenes* growth and proliferation in a dose-dependent manner. The lowest concentration of 4-HPCA that totally inhibited the growth of *L. monocytogenes* was 15.61 mmol/L (after treatment for 24h) [54]. Cueva et al. found that 4-HPCA was active toward *S. aureus*, *E. coli*, *Lactobacillus paraplantarum*, and *Lactobacillus coryniformis*. They showed that treatment with 1000 mg/mL 4-HPCA inhibited *Lactobacillus paraplantarum* growth by 92.94 % [55]. Other study of Samsonowicz et al. [24], indicated that 3-HPAA has stronger antibacterial activity against *B. subtilis*, *P. aeruginosa*, *E. coli*, and *K. oxytoca* than sodium and potassium salts. For example, Na 3-HPA and K 3-HPA inhibited the growth of *E.coli* after 24 h by 44% and 55%, respectively, while for acid alone higher result was obtained (98%) [24]. 

Results of cluster analysis are presented in Figure 11 Based on this, we can group the tested microorganisms according to similar effect to individual treatments. For example, the similar relative cell viability level was observed for *B. subtilis* and *C. albicans* incubation with 2-HPAA (after 24h). Comparable results were noted for the viability level of *E. coli* and *K. aerogenes* cells incubated 48 h with Li 2-HPA. 48 h treatment of *P. fluorescens* and *K. aerogenes* with Na 2-HPA also gives a similar inhibitory effect on their cell viability. 

## 4. Conclusions

In this work, the influence of alkali metals on the biological activity of 2-HPAA and its electronic charge distribution using FT-IR, ^1^H, ^13^C NMR, and UV-VIS spectroscopic methods, thermogravimetric analysis and quantum chemical calculations were discussed. Substitution of the alkali metal ions to the carboxylic group of 2-HPAA caused some characteristic changes in the FT-IR, ^1^H and ^13^C NMR spectra and in geometrical and electronic parameters of salts compared with 2-HPAA. 

Alkali metal ions have been found to influence the distribution of electronic charge around some hydrogen and carbon atoms. This is evidenced by the differences in chemical shifts observed in the ^1^H and ^13^C NMR spectra. Substitution of an alkali metal ion in a -COO^−^ group of acid induces a decrease in the electron charge around C2, C5, C6, C10, and C13 atoms, and an increase around C1, C3, and C4 atoms. In the ^1^H NMR spectra, the greatest changes in the distribution of the electronic charge were observed around the H11 and H12 atoms (from the CH_2_ group) as well as H19 (from the hydroxyl group). The biggest changes concern the charge distribution in the carboxylic group.

Analysis of both experimental and theoretical data shows that the formation of alkali metal salts with 2-hydroxyphenylacetic acid occurs by substituting a metal ion instead a hydrogen atom in the carboxyl group. This is evidenced by the lack of bands from this group in the FT-IR spectra of the salt and the lack of a signal from the hydrogen proton of the carboxyl group in the ^1^H NMR spectra of the salt, as well as changes in the bond length, size of the angles, and the atomic charge distribution of this group. 

It has been found that 2-HPAA and its alkali metal salts with Li, Na, and K in tested concentrations did not show significant antibacterial properties on the tested bacteria (*E. coli*, *K. aerogenes*, *B. subtilis*, and *P. fluorescens*). However, in our study, tested salts were found to be moderate antifungal agents. 

Moreover, alkali metal salts of 2-HPAA demonstrate better antioxidant properties compared to acid (in the ABTS and CUPRAC tests). This was also confirmed by the calculated values of parameters of chemical reactivity. The acid was characterized by a higher chemical hardness value than the salts (the differences between the acid and the salts are: 0.366 eV, 0.7575 eV, and 0.706 eV for the salts Li, Na, and K, respectively. Additionally, smaller differences in energy between the HOMO and LUMO orbitals in salts compared to the acid indicate their greater antioxidant reactivity, whereas 2-hydroxyphenylacetic acid should be chemically less active than alkali metal salts.

## Figures and Tables

**Table 1:** The wavenumbers, intensities, and assignments of the chosen bands from the FT-IR spectra of 2-hydroxyphenylacetic acid and lithium, sodium, potassium, cesium, and rubidium 2-hydroxyphenylacetates.

2-HPAA	Li 2-HPA	Na 2-HPA	K 2-HPA	Rb 2-HPA	Cs 2-HPA	Assignments	No. of the Aromatic Ring Vibrative According to [41]
		3280s		3284m	3405m	ν(OH)_ar_	
3372vs						ν(OH)	
3191s						ν(CH)	2
	3063s	3065m	3058m	3059s		ν(CH)	20b
	2960s		3012m	3012m	3015m	ν(CH)	20a
2947m	2892s	2927m	2964s	2964m		ν_ar_(CH_2_)	
	2730s	2857m	2910m			ν_s_(CH_2_)	
1694vs						ν(C=O)	
1603s	1588vs	1590s		1592s	1592s	ν(CC)	8b
	1573vs	1564vs	1575vs	1575vs	1565vs	ν_as_(COO^−^)	
1508m		1504m	1508vs	1507s	1505m	ν(CC)	19a
1462s	1467m	1457vs	1436vs	1436s	1433s	ν(CC)	19b
1385vs						β(OH)	
	1392s	1407s	1382vs	1381s	1385s	ν_as_(COO^−^)	
1354s						β(CH)	3
		1364s				ν(CC)	14
	1324m	1319m	1314m	1314m	1314m	γ(CH_2_)	
1307vs						ν(C-OH)	
1238s	1256s	1258s	1262s	1262m	1260m	ν(CH)	13
1178m						ν(CH)	7a
	1190w	1200m			1200w	β(CH)	9a
	1180w	1185m	1178w	1178w	1184m	β(CH)	9b
1098m	1157w					β(CH)	18a
1040m	1102s	1105s	1103w	1103w	1103w	β(CH)	18b
	1037w	1044w	1041w	1041w	1042w	α(CCC)	12
935w						γ(CH)	5
	955m	950w	955s	953m		β_as_(CH_2_)	
872m						ν(C-COOH)	
	938w	943w				γ(CH)	
	865m	879m	882w	881w	877w	β_s_(COO^−^)	
783m						β(C=O)	
	843w	855m	851w	851w	855w	γ(CH)	
759s	786w		791m	792m		γ(CH)	11
	752s	746s	744s	744s	749s	γ_s_(COO^−^)	
676m	708m	694s	717m	715w	716w	φ(CC)	4
	635w	633w	644m	644m	693m	β_as_(COO^−^)	
622s						γ(C=O)	
592m	585w	585w	585w	572w		α(CCC)	6a
541m						φ(CC)	16b
	516w	469w	479w	479w	477w	α(CCC)	6b
		381w	394w	394w	395w	φ(CC)	16a

Note: ν—stretching vibrations, α—the aromatic ring in-plane bending, β—bending in-plane, γ—bending out-of-plane, and φ—the aromatic ring out-of-plane bending. Band intensity: s—strong; m—medium; w—weak; and vw—very weak.

**Table 2:** Comparison of spectral parameters (stretching and deformation vibrations of the carboxylate anion) from the FT-IR spectra of 2-HPAA alkali metal salts.

Compound	Wavenumber/cm^−1^					
	ν_s_(COO^−^)	ν_as_(COO^−^)	Δν = ν_as_(COO^−^)―ν_s_(COO^−^)	β_s_(COO^−^)	β_as_(COO^−^)	Δβ = β_as_(COO^−^)―β_s_(COO^−^)
Li 2-HPA	1392	1573	181	865	635	230
Na 2-HPA	1407	1564	157	879	633	246
K 2-HPA	1382	1575	193	882	644	238
Rb 2-HPA	1381	1575	194	881	644	237
Cs 2-HPA	1385	1565	180	877	693	184

**Table 3:** Calculated parameters of chemical reactivity of phenylacetic acid (PAA), 2-hydroxyphenylacetic acid (2-HPAA) and its chosen alkali metal salts.

Parameter	PAA	2-HPAA	Li 2-HPA	Na 2-HPA	K 2-HPA
Energy (Hartree *)	−460.2682 **	−535.5167	−542.5134	−697.2753	−1134.9197
Dipole moment (Debye)	1.3872 **	1.4556	3.6183	6.0303	7.5919
E_HOMO_ [eV]	−6.65 **	−6.4230	−6.0310	−5.7810	−5.6110
E_LUMO_ [eV]	−0.01 **	−0.6520	−0.9920	−1.5250	−1.2520
ΔE_(LUMO-HOMO)_ [eV]	6.64 **	5.7710	5.0390	4.2560	4.3590
Ionization potential (IP) [eV]	6.65	6.4230	6.0310	5.7810	5.6110
Electronaffinity (A) [eV]	0.01	0.6520	0.9920	1.5250	1.2520
Electronegativity (χ) [eV]	3.33	3.5375	3.5115	3.6530	3.4315
Electronic chemical potential (μ) [eV]	−3.33	−3.5375	−3.5115	−3.6530	−3.4315
Chemical hardness (η) [eV]	3.32	2.8855	2.5195	2.1280	2.1795
Chemical softness (σ) [eV]	0.151	0.1733	0.1985	0.2350	0.2294
Electrophilicity index (ω) [eV]	1.670	2.1684	2.4470	3.1354	2.7014

* 1 Hartree = 2625.5 kJ/mol; ** taken from [46].

**Table 4:** Thermoanalytical results (TG, DTG, and DSC) for the 2-HPAA and its alkali metal salts (processed under an air atmosphere).

Compound	Stage	TG	DTG(DSC)	Peak Nature	Mass Loss/%		Loss	Final Residue
		T_range_/°C	T_max peaks_/°C		Calculated	Found		
C_8_H_8_O_3_	I	108–217	203(206)	endo	100	98.49	Phenyl, CH_3_COOH	-
Li(C_8_H_8_O_3_)*1/2H_2_O	IIIIIIIV	128–198 198–524 524–553 553–905	162(162) 284(308) 541(544) -	endo exo exo -	5.52 69.97 78.60 90.82	5.78 67.14 79.62 87.93	1/2H_2_O Phenyl CO_2_,H_2_O CO_2_	Li(C_8_H_8_O_3_) LiCH_3_COO Li_2_CO_3_ Li_2_O
Na(C_8_H_8_O_3_)	I	116–595	224(222) 285(291)	-	-	-	-	-
	II	595–848	769(759) 796(797) 812(813)	exo exo exo	69.60	68.78	CO_2_,H_2_O	Na_2_CO_3_
K(C_8_H_8_O_3_)	I	197–534	223(221) 434(440)	exo exo	-	-	Phenyl	KCH_3_COO
	II	534–700	694(697)	exo	56.88	54.50		
	III	700–857	780(780)	exo	63.71	61.85	CO_2_,H_2_O	K_2_CO_3_
Rb(C_8_H_8_O_3_)	I	160–581	216(212) 445(451)	endo exo	48.80	49.47	Phenyl, CO_2_,H_2_O	Rb_2_CO_3_
	II	581–750	632(639)	exo	37.71	38.20	CO_2_	Rb_2_O
Cs(C_8_H_8_O_3_)*1.25 H_2_O	I	36–127	76(76)	endo	7.01	7.17	1.25 H_2_O	Cs(C_8_H_8_O_3_)
	II	127–562	445(445)	exo	57.32	58.00	Phenyl	CsCH_3_COO
	III	562–730	603(604) 718(719)	exo exo	49.49	47.42	CO_2_,H_2_O	Cs_2_CO_3_

**Table 5:** The elemental analysis data of 2-HPAA and its alkali metal salts.

Compound		C/%		H/%	
		Experimental	Calculated	Experimental	Calculated
2-HPAA	C_8_H_8_O_3_	63.16	63.16	5.25	5.30
Li 2-HPA	Li (C_8_H_8_O_3_)*1/2H_2_O	59.98	58.72	4.41	5.50
Na 2-HPA	Na (C_8_H_8_O_3_)	54.08	54.87	4.10	4.61
K 2-HPA	K (C_8_H_8_O_3_)	49.81	50.25	3.75	4.22
Rb 2-HPA	Rb (C_8_H_8_O_3_)	48.25	50.05	3.75	3.98
Cs 2-HPA	Cs (C_8_H_8_O_3_)*1.25 H_2_O	33.58	34.53	2.52	2.90

## Data Availability

The data presented in this study are available on request from the corresponding author.

## Supplementary Materials

The following are available online at https://www.mdpi.com/article/10.3390/ma14247824/s1, Table S1: Proton 1H and carbon 13C shifts for 2-hydroxyphenylacetic acid and 2-hydroxyphenylacetates, Table S2: The selected bond lengths (Å) and angles (o) between bonds in 2-HPAA and its alkali metal 2-hydroxyphenylacetates, Table S3: Data of NBO and Mulliken atomic charge analysis for 2-HPAA and its alkali metal 2-hydroxyphenylacetates, Table S4: Change of electron charge in the carboxylic anion -COO− depending on the metal attached (calculated by NBO and Mulliken method), Figure S1: The FT-IR spectra of 2-HPAA and its alkali metal 2-hydroxyphenylacetates, Figure S2: The 1H NMR spectra of 2-HPAA (a) and its alkali metal salts (b–f), Figure S3: The 13C NMR spectra of 2-HPAA (a) and its alkali metal 2-hydroxyphenylacetates (b–f).
