# Supplementary material for: Spectroscopic, Thermal, Microbiological, and Antioxidant Study of Alkali Metal 2-Hydroxyphenylacetates
Source: Materials (Basel). 2021 Dec 17;14(24):7824. doi: 10.3390/ma14247824 (PMC8708765; doi:10.3390/ma14247824)
Supplement: Supplementary file 1 [file materials-14-07824-s001.zip › materials-1428695-SI.pdf]

Supplementary

Spectroscopic, Thermal, Microbiological, and Antioxidant Study of Alkali metal 2-Hydroxyphenylacetates

Mariola Samsonowicz [1,*], Ewelina Gołębiewska [1], Elżbieta Wołejko [1], Urszula Wydro [1], Grzegorz Świderski [1], Joanna Zwolińska [2], Monika Kalinowska [1] and Włodzimierz Lewandowski [1]

[1] Department of Chemistry, Biology and Biotechnology, Institute of Civil Engineering and Energetics, Faculty of Civil Engineering and Environmental Science, Bialystok University of Technology, Wiejska 45E Street, 15-351 Bialystok, Poland; e.golebiewska@pb.edu.pl (E.G.); e.wolejko@pb.edu.pl (E.W.); u.wydro@pb.edu.pl (U.W.), g.swiderski@pb.edu.pl (G.S.); m.kalinowska@pb.edu.pl (M.K.); w-lewando@wp.pl (W.L.)

[2] Centre for Advanced Technologies, Adam Mickiewicz University, Uniwersytetu Poznańskiego 10 Street, 61-614 Poznań, Poland; joakol1@amu.edu.pl

* Correspondence: m.samsonowicz@pb.edu.pl

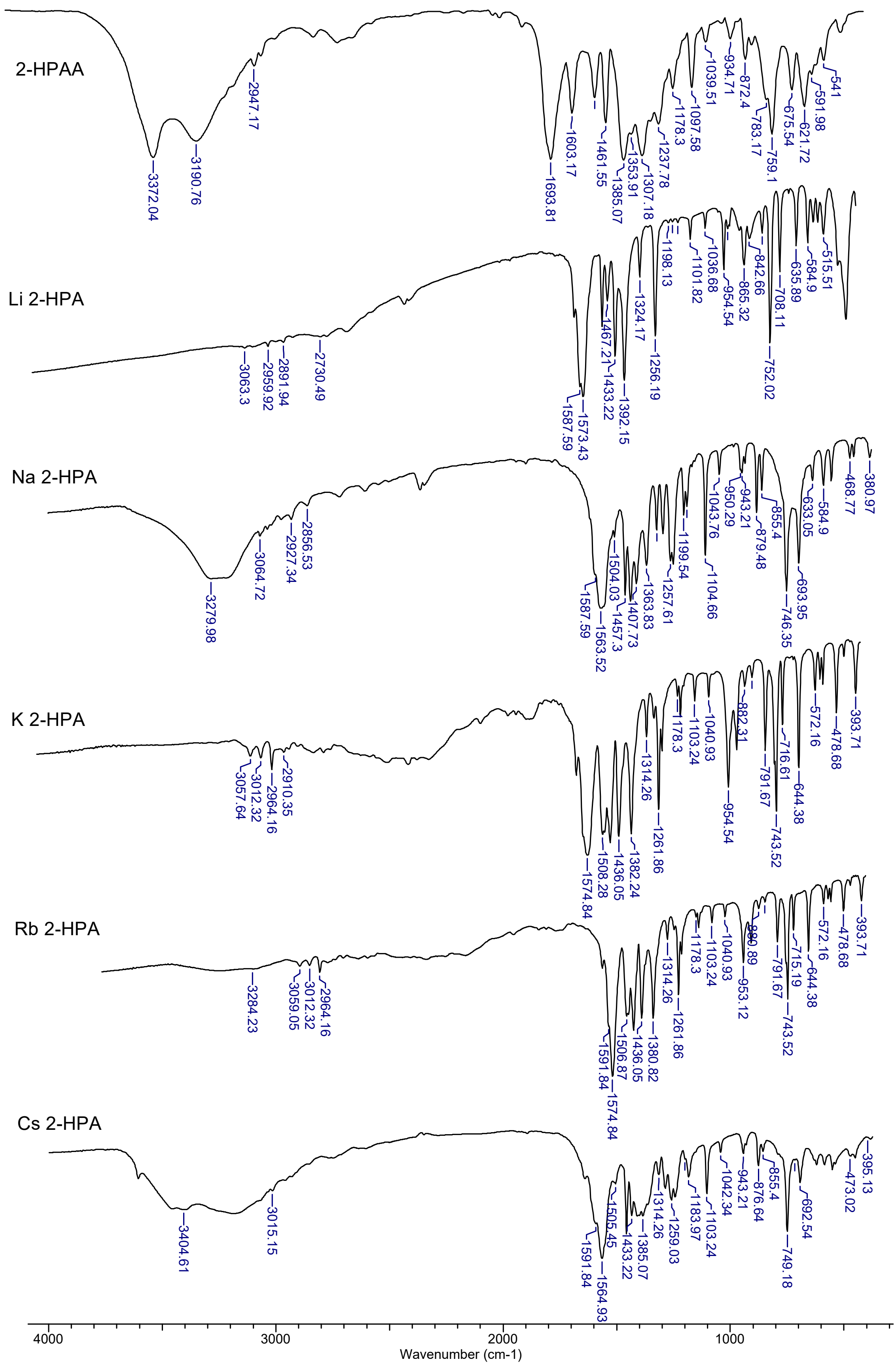

Figure S1. The FT-IR spectra of 2-HPAA and its alkali metal 2-hydroxyphenylacetates.

(**a**)

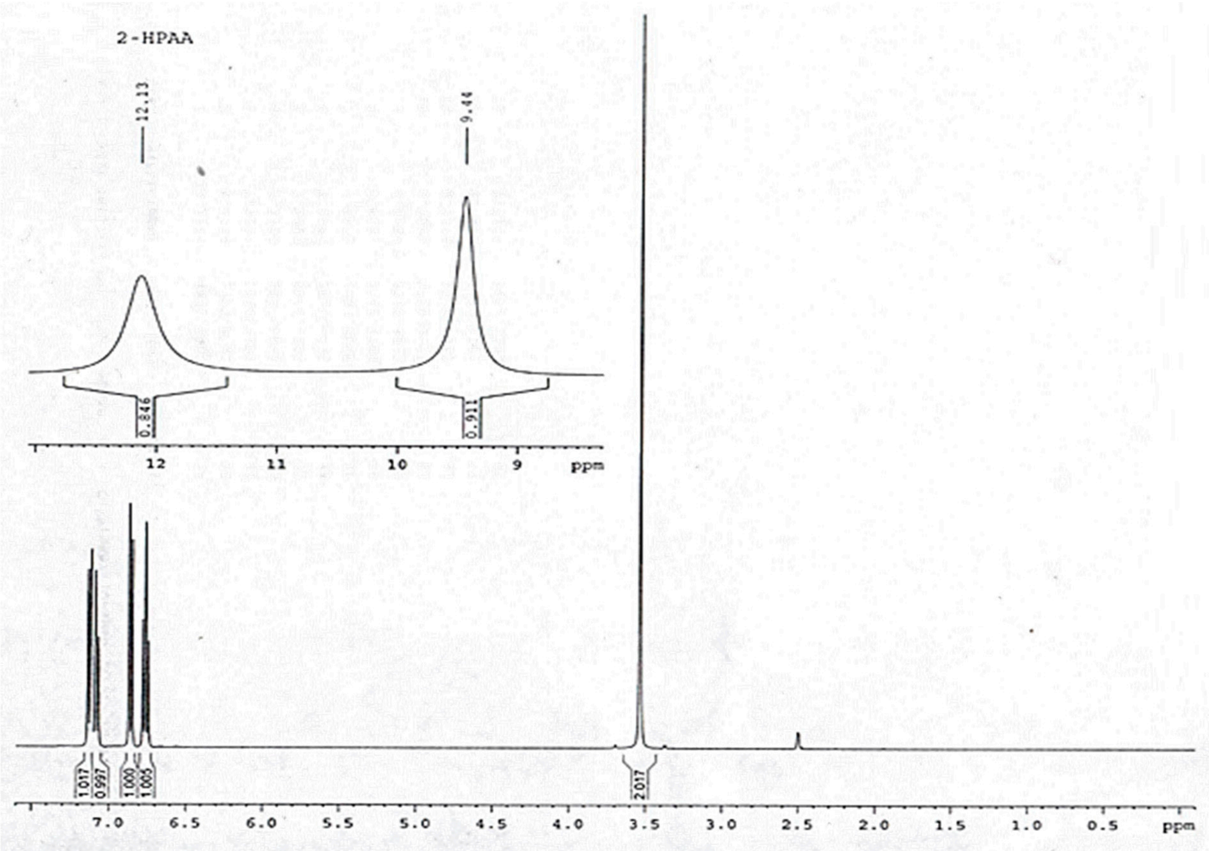
2-HPAA
12.13
9.44
0.846
0.911
12
11
10
9
ppm
1.017
0.997
1.000
1.005
2.017
7.0
6.5
6.0
5.5
5.0
4.5
4.0
3.5
3.0
2.5
2.0
1.5
1.0
0.5
ppm

(**b**)

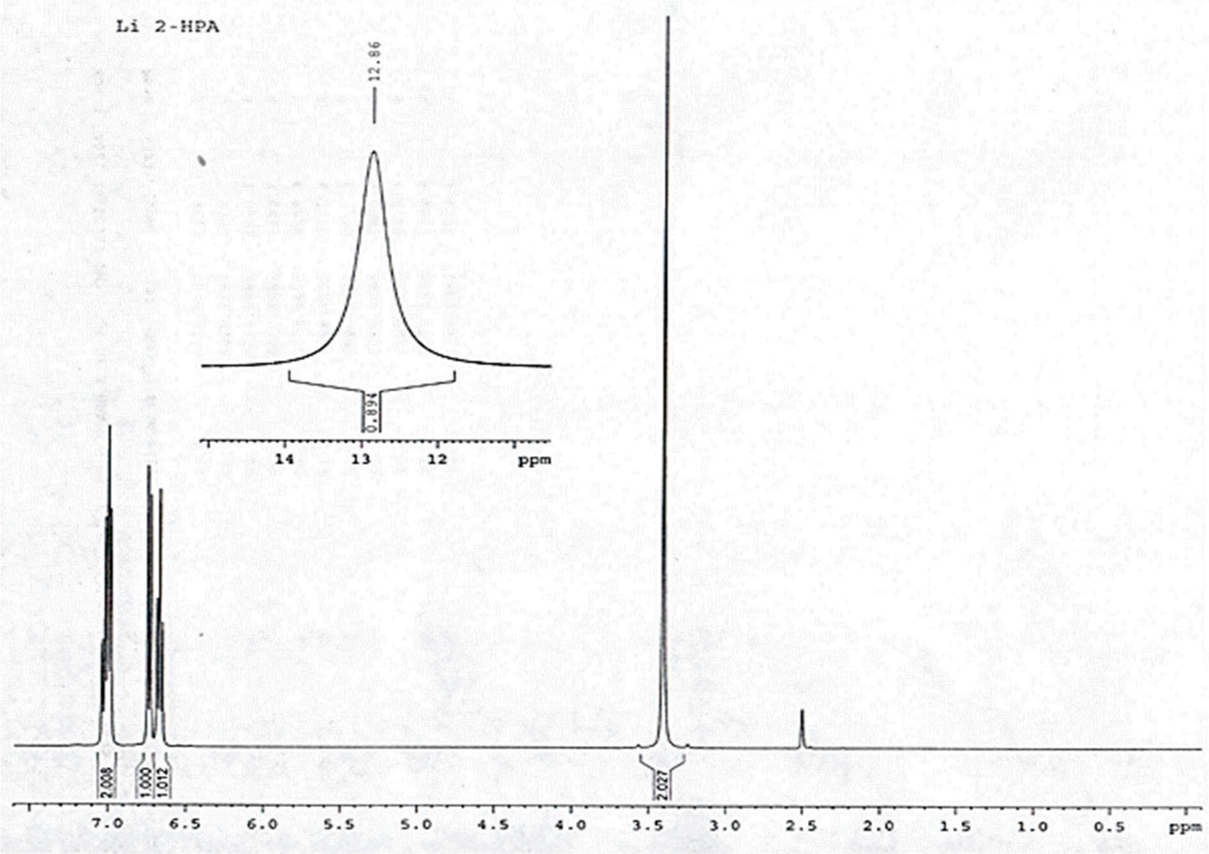
Li 2-HPA
12.86
0.894
14
13
12
ppm
2.008
1.000
1.012
2.027
7.0
6.5
6.0
5.5
5.0
4.5
4.0
3.5
3.0
2.5
2.0
1.5
1.0
0.5
ppm

(c)

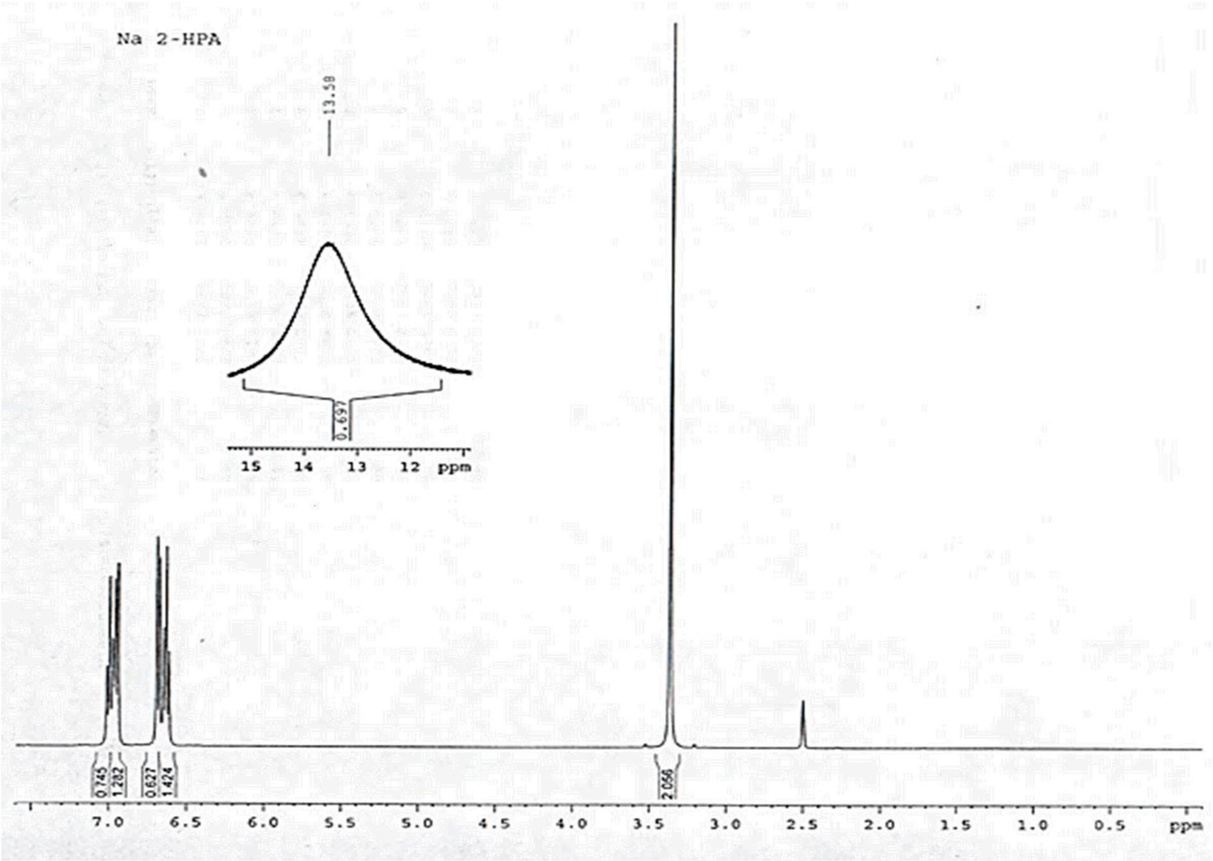

Na 2-HPA
13.58
0.697
15
14
13
12
ppm
0.745
1.282
0.627
1.424
2.056
7.0
6.5
6.0
5.5
5.0
4.5
4.0
3.5
3.0
2.5
2.0
1.5
1.0
0.5
ppm

(d)

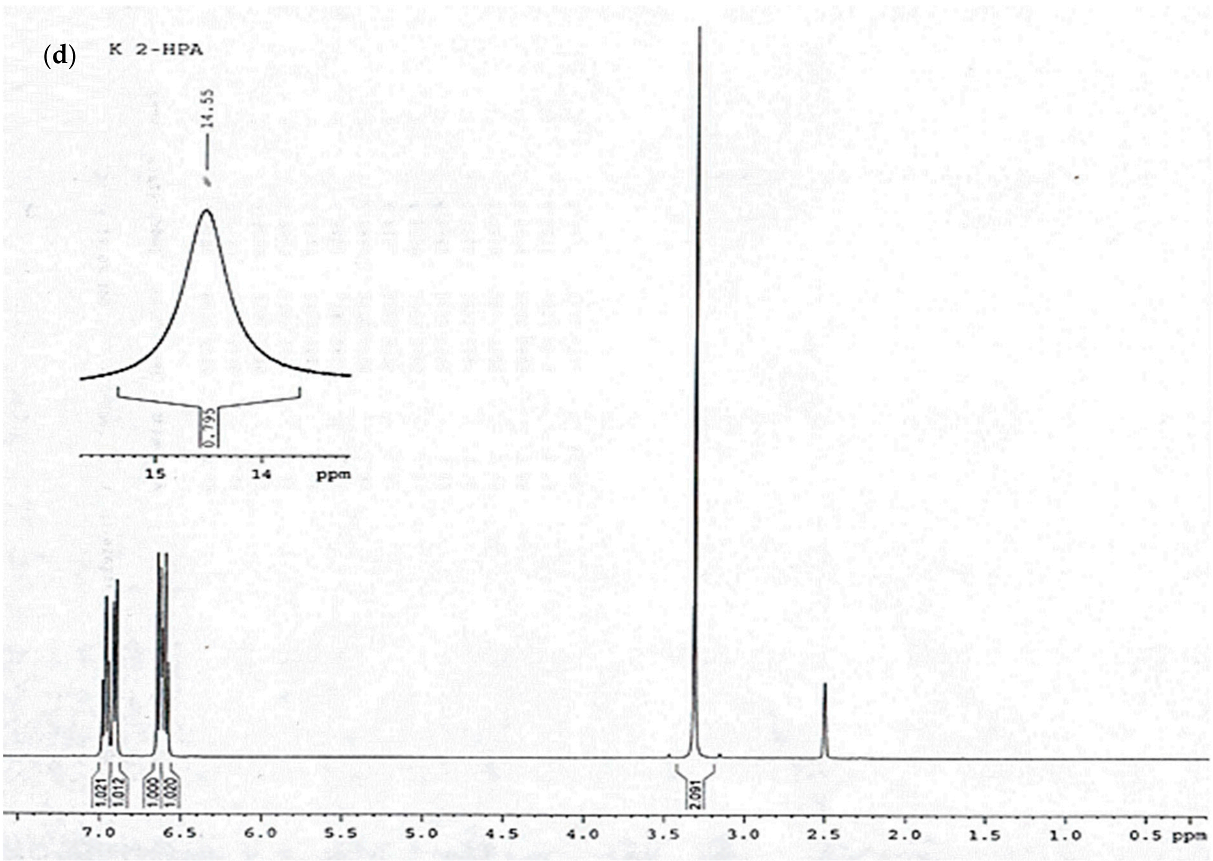

K 2-HPA
14.55
0.795
15
14
ppm
1.021
1.012
1.000
1.020
2.091
7.0
6.5
6.0
5.5
5.0
4.5
4.0
3.5
3.0
2.5
2.0
1.5
1.0
0.5 ppm

(**e**)

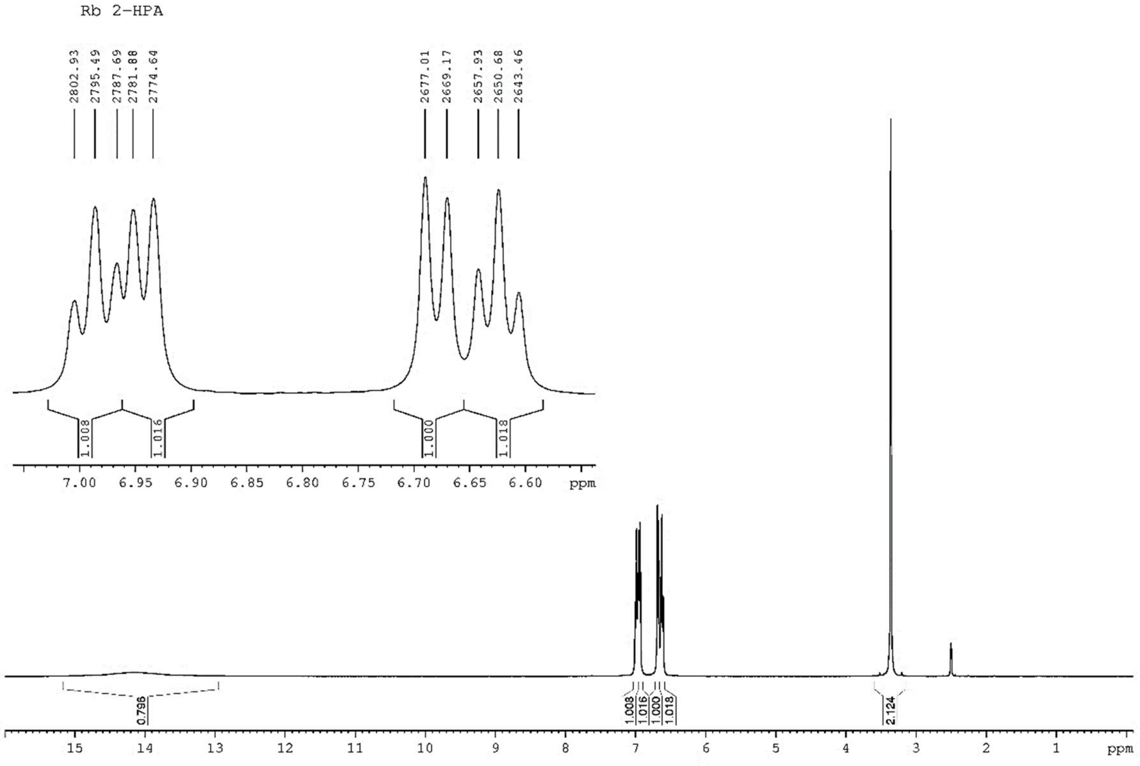

(**f**)

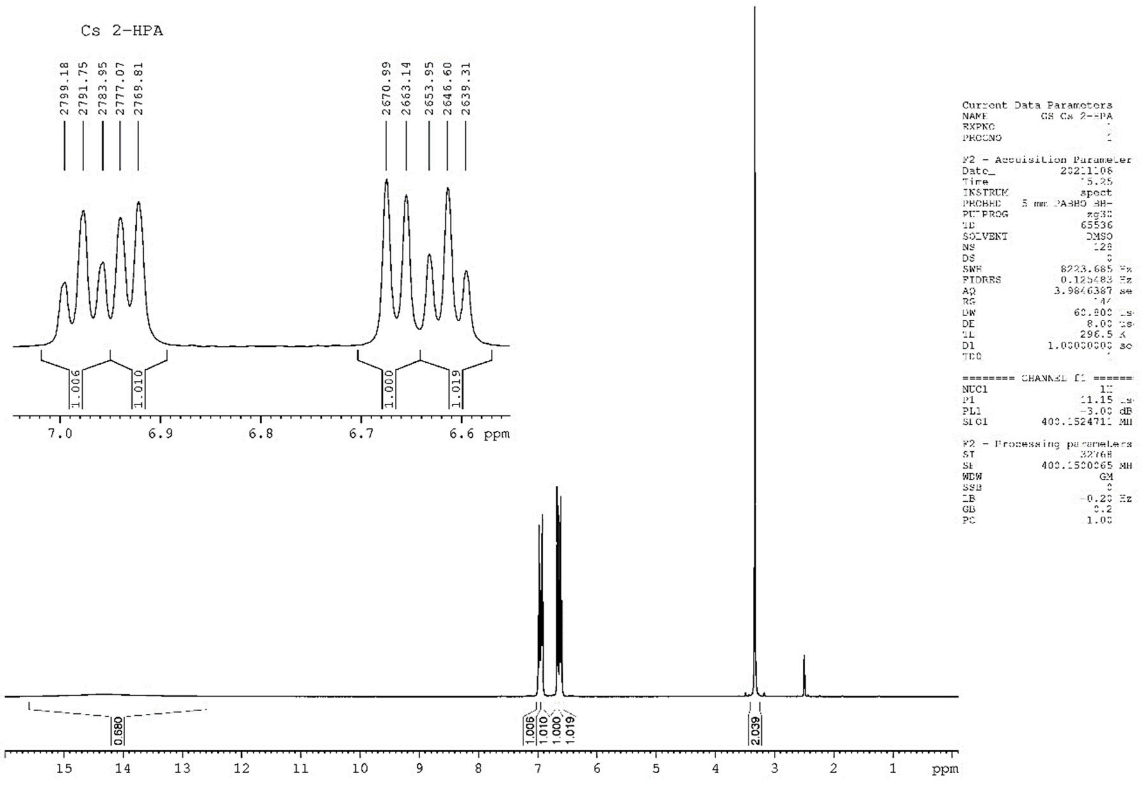

Figure S2. The ^{1}H NMR spectra of 2-HPAA (**a**) and its alkali metal salts (**b**–**f**).

(a)

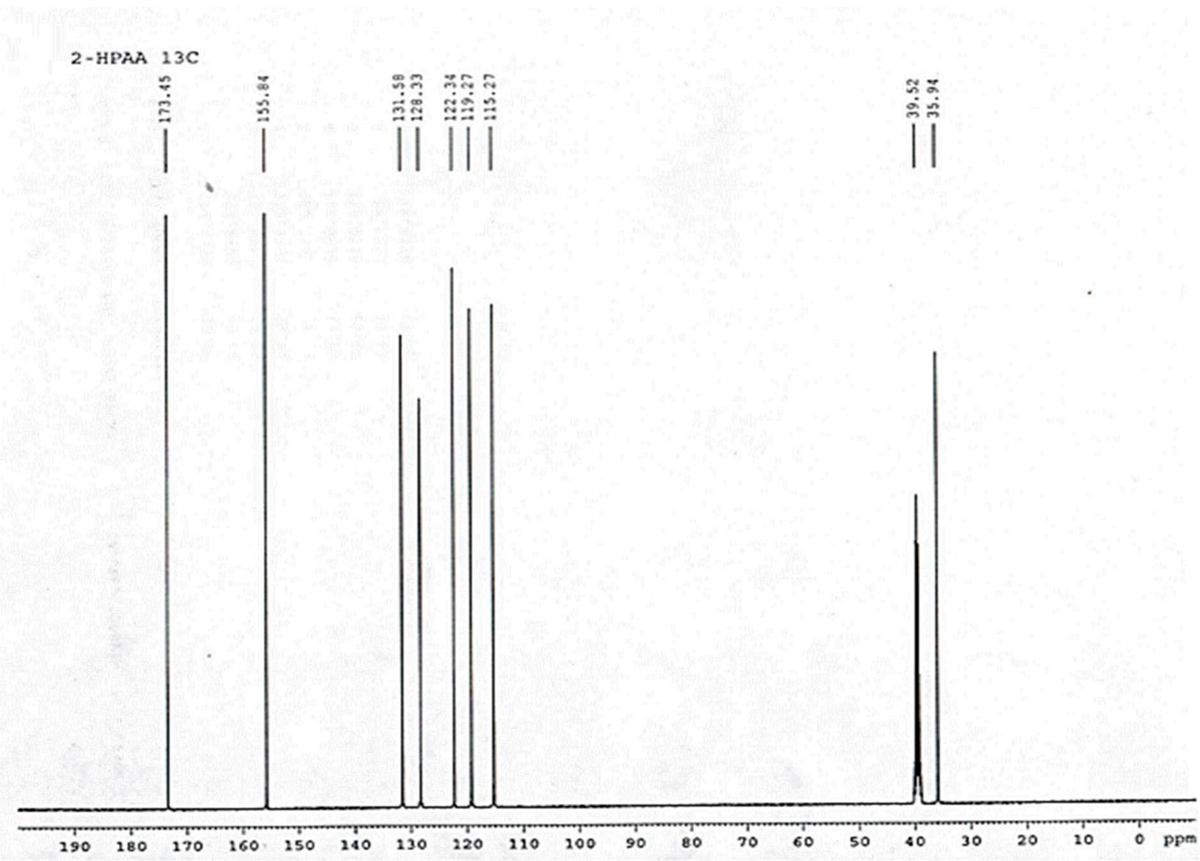

2-HPAA 13C
173.45
155.84
131.58
128.33
122.34
119.27
115.27
39.52
35.94
190
180
170
160
150
140
130
120
110
100
90
80
70
60
50
40
30
20
10
0
ppm

(b)

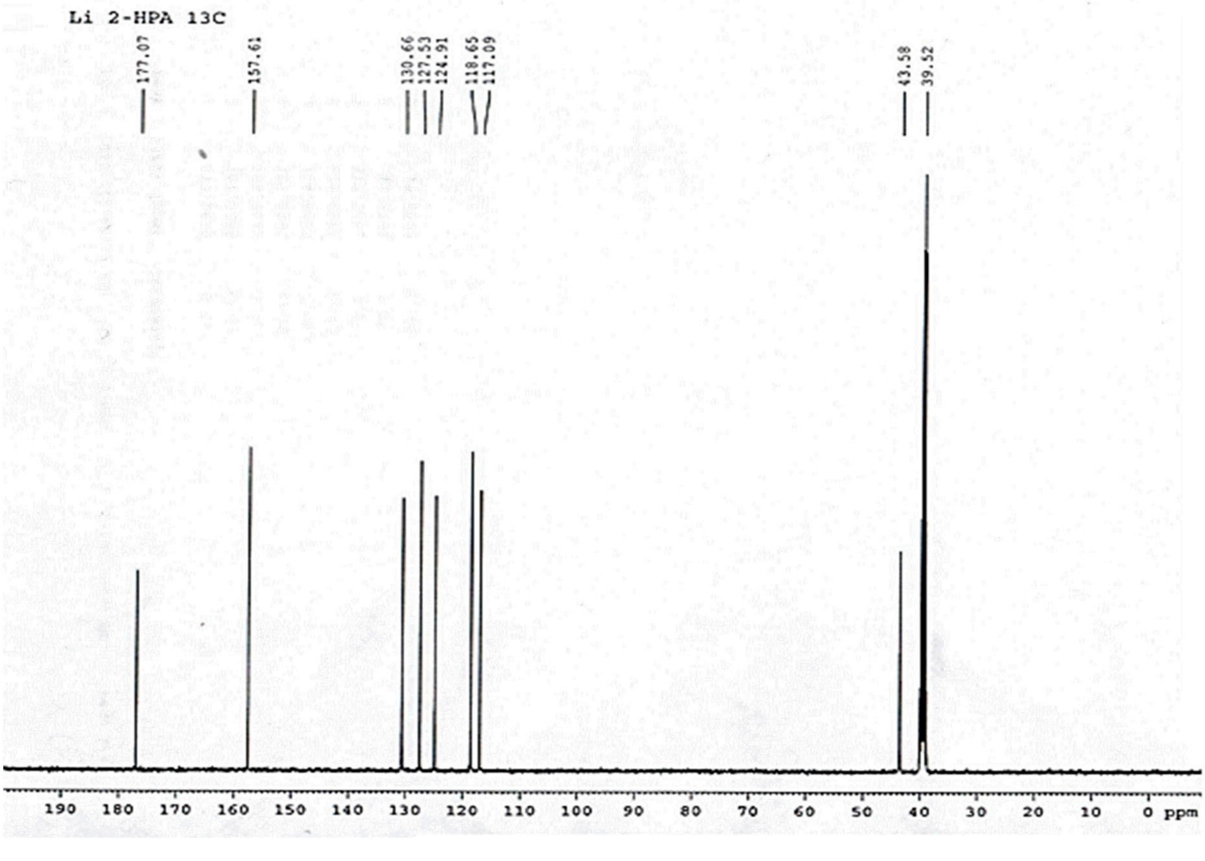

Li 2-HPA 13C
177.07
157.61
130.66
127.53
124.91
118.65
117.09
43.58
39.52
190
180
170
160
150
140
130
120
110
100
90
80
70
60
50
40
30
20
10
0
ppm

(c)

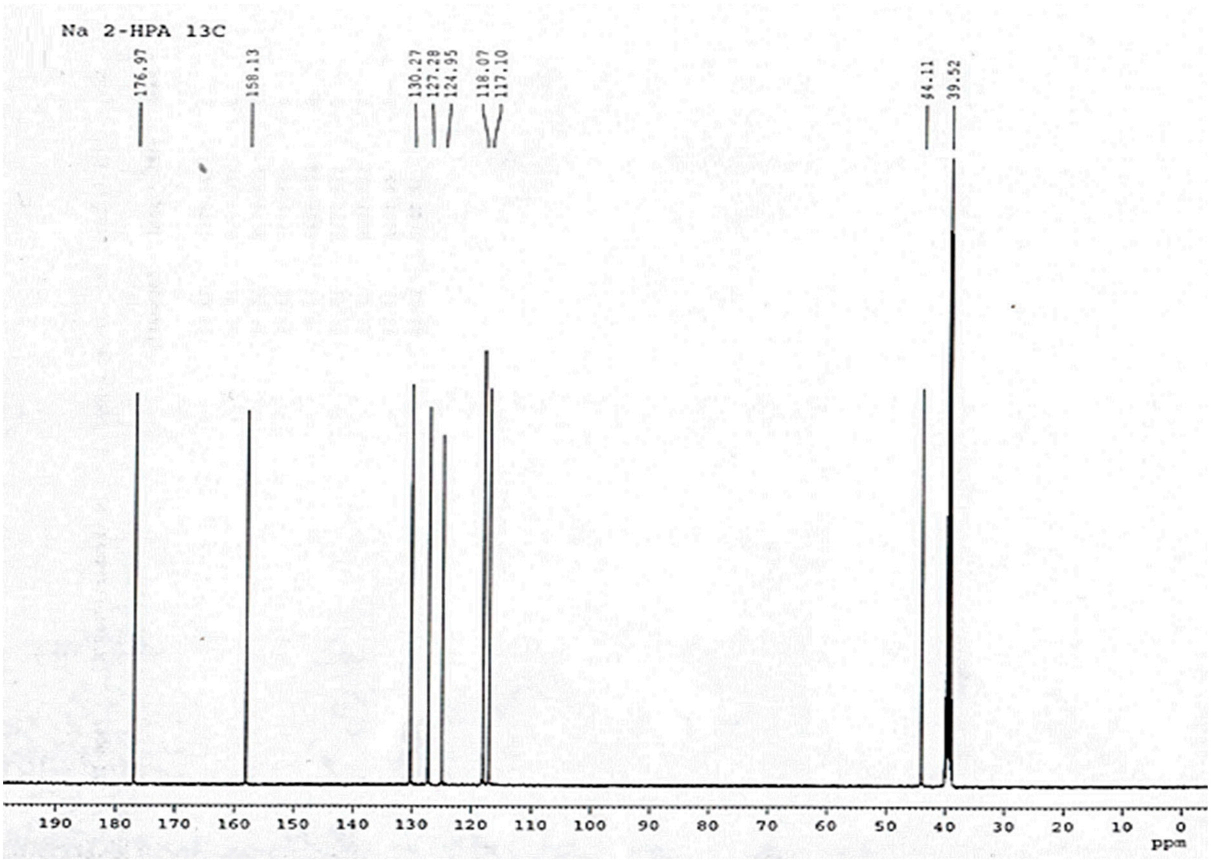
Na 2-HPA 13C
176.97
158.13
130.27
127.28
124.95
118.07
117.10
44.11
39.52
190 180 170 160 150 140 130 120 110 100 90 80 70 60 50 40 30 20 10 0
ppm

(d)

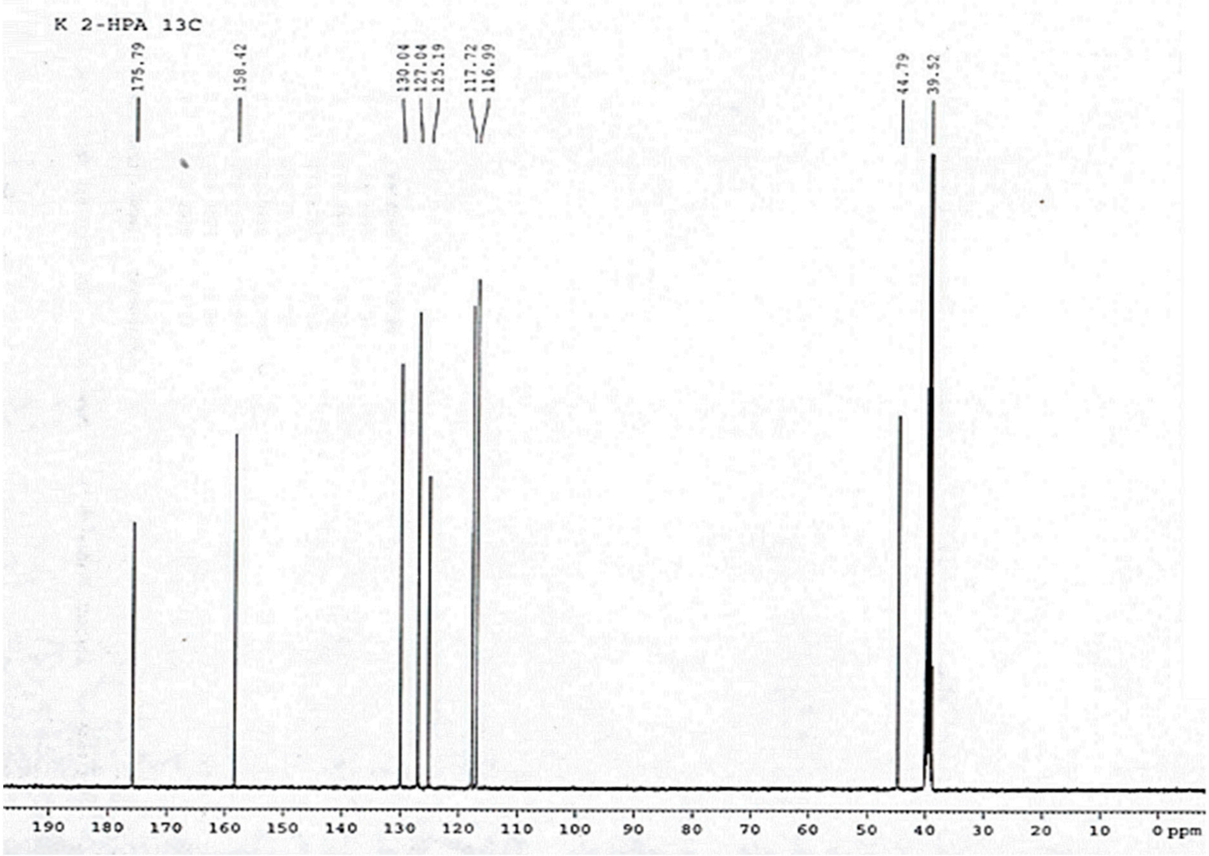
K 2-HPA 13C
175.79
158.42
130.04
127.04
125.19
117.72
116.99
44.79
39.52
190 180 170 160 150 140 130 120 110 100 90 80 70 60 50 40 30 20 10 0 ppm

(e)

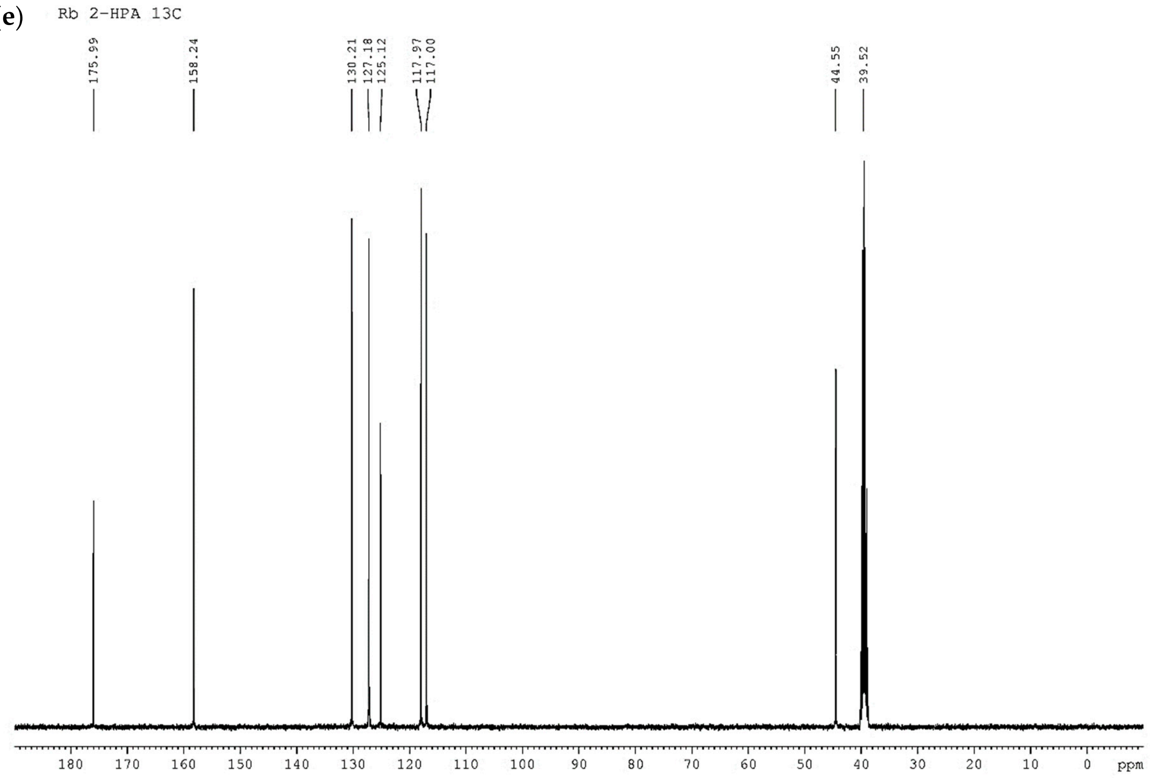

(f)

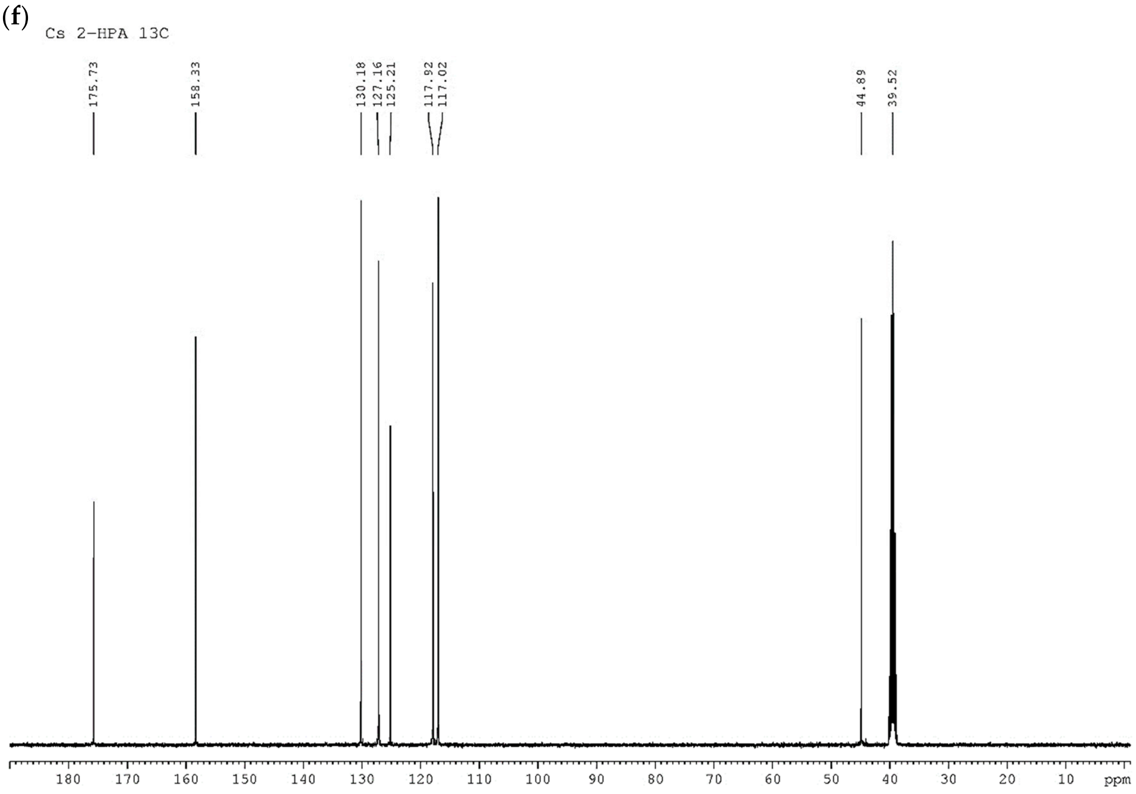

Figure S3. The ^{13}C NMR spectra of 2-HPAA (**a**) and its alkali metal 2-hydroxyphenylacetates (**b**–**f**).

Table S1. Proton ^1H and carbon ^{13}C shifts for 2-hydroxyphenylacetic acid and 2-hydroxyphenylacetates.

	**2-HPAA**	**Li 2-HPA**	**Na 2-HPA**	**K 2-HPA**	**Rb 2-HPA**	**Cs 2-HPA**
			^1H NMR			
H(16)	12.13	12.86	13.58	14.55	14.15	14.33
H(19)	9.44	-	-	-	-	-
H(7)	7.12	7.02	7.01	6.98	7.00	6.99
H(9)	7.08	6.99	6.95	6.95	6.97	6.96
H(8)	6.86	6.74	6.68	6.63	6.68	6.66
H(17)	6.76	6.67	6.63	6.60	6.62	6.61
H(11, 12)	3.53	3.41	3.37	3.31	3.36	3.34
			^{13}C NMR			
C(13)	173.45	177.07	176.97	175.79	175.99	175.73
C(5)	155.86	157.61	158.13	158.42	158.24	158.33
C(3)	131.58	130.66	130.27	130.04	130.21	130.18
C(1)	128.33	127.53	127.28	127.04	127.18	127.16
C(2)	122.34	124.91	124.95	125.19	125.12	125.21
C(4)	119.27	118.65	118.07	117.72	117.97	117.92
C(6)	115.27	117.08	117.10	116.99	117.00	117.02
C(10)	39.52	43.58	44.11	44.79	44.54	44.89

Table S2. The selected bond lengths (Å) and angles (o) between bonds in 2-HPAA and its alkali metal 2-hydroxyphenylacetates.

**Atom Numbers***	**2-HPAA**	**Li 2-HPA**	**Na 2-HPA**	**K 2-HPA**
		**Bond Lengths (Å)**		
C10-C13	1.519	1.529	1.539	1.543
C13-O14	1.203	1.268	1.263	1.261
C13-O15	1.359	1.273	1.268	1.267
O15-H16/M	0.969	1.853	2.207	2.154
O14-H16/M	2.290	1.857	2.208	2.516
		**Angles (°)**		
C3-C4-C5	118.14	117.74	117.55	117.13
C4-C10-C13	111.10	115.58	115.81	115.81
C10-C13-O14	126.92	120.88	119.68	119.35
C10-C13-O15	110.55	117.81	116.42	116.07
C13-O15-H16/M	106.98	82.72	87.62	91.21
C13-O14-H16/M	55.30	82.67	87.66	91.27

* Atom numbers according to Figure 2.

Table S3. Data of NBO and Mulliken atomic charge analysis for 2-HPAA and its alkali metal 2-hydroxyphenylacetates.

Atom	Charge (e[1])							
	2-HPAA		Li 2-HPA		Na 2-HPA		K 2-HPA	
	**NBO**	**Mulliken**	**NBO**	**Mulliken**	**NBO**	**Mulliken**	**NBO**	**Mulliken**
C1	−0.182	−0.188	−0.190	−0.219	−0.194	−0.235	−0.196	−0.233
C2	−0.227	−0.439	−0.229	−0.434	−0.231	−0.445	−0.231	−0.456
C3	−0.169	−0.418	−0.171	−0.381	−0.172	−0.317	−0.173	−0.300
C4	−0.076	0.913	−0.064	0.960	−0.058	1.023	−0.054	1.033
C5	0.327	−0.250	0.325	−0.274	0.324	−0.383	0.323	−0.472
C6	−0.274	−0.173	−0.278	−0.184	−0.280	−0.191	−0.821	−0.199
H7	0.205	0.157	0.202	0.150	0.201	0.146	0.200	0.144
H8	0.206	0.164	0.203	0.159	0.201	0.156	0.200	0.154
H9	0.202	0.145	0.199	0.146	0.198	0.144	0.197	0.140
H10	−0.500	−0.212	−0.497	0.012	−0.497	0.093	−0.500	−0.009
H11	0.236	0.211	0.228	0.192	0.222	0.183	0.219	0.173
H12	0.248	0.250	0.242	0.243	0.238	0.231	0.235	0.221
C13	0.817	−0.197	0.785	−0.132	0.784	−0.189	0.786	−0.334
O14	−0.591	−0.259	−0.828	−0.300	−0.815	−0.434	−0.818	−0.467
O15	−0.695	−0.157	−0.844	−0.329	−0.830	−0.452	−0.834	−0.485
H16/M	0.482	0.274	0.933	0.223	0.931	0.517	0.952	0.940
H17	0.161	0.131	0.196	0.125	0.194	0.261	0.193	0.258
O18	−0.681	−0.225	−0.680	−0.224	−0.680	−0.227	−0.680	−0.226
H19	0.471	0.272	0.466	0.266	0.464	0.121	0.463	0.118

[1] e = $1.6021892 \cdot 10^{-19}$.

Table S4. Change of electron charge in the carboxylic anion -COO^- depending on the metal attached (calculated by NBO and Mulliken method).

Atom	Charge (e[1])							
	2-HPAA		Li 2-HPA		Na 2-HPA		K 2-HPA	
	**NBO**	**Mulliken**	**NBO**	**Mulliken**	**NBO**	**Mulliken**	**NBO**	**Mulliken**
C13	0.817	−0.197	0.785	−0.132	0.784	−0.189	0.786	−0.334
C14	−0.591	−0.259	−0.828	−0.300	−0.815	−0.434	−0.818	−0.467
C15	−0.695	−0.157	−0.844	−0.329	−0.830	−0.452	−0.834	−0.485
-COO^-	−0.469	−0.613	−0.887	−0.761	−0.861	−1.075	−0.866	−1.286
